# Implementation of a ‘Joint Clinic’ to resolve unmet need for orthopaedic services in patients with hip and knee osteoarthritis: a program evaluation

**DOI:** 10.1186/s12891-019-2702-1

**Published:** 2019-07-12

**Authors:** J. Haxby Abbott, Aimee L. Ward, Chris Crane, Catherine M. Chapple, Kirsten Stout, Liam Hutton, Virginia Martin, Helen Harcombe, Daniel Cury Ribeiro, David Gwynne Jones

**Affiliations:** 1 Centre for Musculoskeletal Outcomes Research, Dunedin School of Medicine, University of Otago, Dunedin, New Zealand; 2 Southern District Health Board, Dunedin, New Zealand; 3 Centre for Health Activity and Rehabilitation Research, School of Physiotherapy, University of Otago, Dunedin, New Zealand; 4 Department of Preventive and Social Medicine, University of Otago, Dunedin, New Zealand

**Keywords:** Orthopedics, Osteoarthritis Knee, Osteoarthritis Hip, Osteoarthritis/Therapy, Referral and consultation, Secondary care, Primary health care, Program evaluation

## Abstract

**Background:**

Osteoarthritis is the most common form of arthritis, principally affecting the older population. Highly prevalent, disabling diseases such as osteoarthritis strain the capacity of health systems, and can result in unmet need for services. The Joint Clinic was initiated to provide secondary care consultations and access to outpatient services for people with advanced hip or knee osteoarthritis, who were referred by their general practitioner for orthopaedic consultation but not offered an orthopaedic specialist appointment.

**Methods:**

This longitudinal programme evaluation comprised four components: a proof-of-concept evaluation; an implementation evaluation; a process evaluation; and an outcomes evaluation. Interviews and surveys of general practitioners, staff, and patients were conducted pre- and post-implementation. Interviews were transcribed, and thematic analysis was completed. In addition, Joint Clinic patient visits and outcomes were reviewed.

**Results:**

One hundred and eleven primary care physicians (GPs) and 66 patients were surveyed, and 28 semi-structured interviews of hospital staff and GPs were conducted. Proof of concept was satisfied. Interim and final implementation evaluations indicated adherence to the concept model, high levels of acceptance of and confidence in the programme and its staff, and timely completion within budget. Process evaluation revealed positive impacts of the programme and positive stakeholder perceptions, with some weaknesses in communication to the outer context of primary care. The Joint Clinic saw a total of 637 patient visits during 2 years of operation. Unmet need was reduced by 90%. Patient and referring physician satisfaction was high. Hospital management confirmed that the programme will continue.

**Conclusions:**

This evaluation indicates that the Joint Clinic concept model is fit for purpose, functioned well within the organisation, and achieved its primary objective of reducing unmet need of secondary care consultation for those suffering advanced hip or knee osteoarthritis.

**Electronic supplementary material:**

The online version of this article (10.1186/s12891-019-2702-1) contains supplementary material, which is available to authorized users.

## Background

Osteoarthritis (OA) is the most common form of arthritis, principally affecting the older population. The Global Burden of Diseases, Injuries, and Risk Factors Study 2015 found that the prevalence of OA increased 32% between 2005 and 2015 [1]. The high prevalence and increasing disability burden of OA mean it is a high priority condition, and has been formally recognised as such by the World Health Organisation (WHO) [2].

Many health systems worldwide will need to adapt to a higher proportion of older people as population demographics change. In New Zealand, those over the age of 65 years will make up over one quarter of the population by the late 2030’s [3]. Osteoarthritis of the hip and knee is the most common condition for which joint replacements are indicated, and as the population ages, demand for joint replacement surgery is predicted to rise significantly [3]. This scenario will place significant stress on the health resources in New Zealand. The Southern District Health Board (SDHB), the public health services provider for Dunedin, New Zealand, has seen a substantial rise in demand for joint replacement surgery, and a shortfall of orthopaedic specialist resources to meet the demand of general medical practitioner (GP) referrals for patients with osteoarthritis [4, 5]. This has resulted in a growing unmet need for secondary care consultations and OA management. A report by the SDHB general practitioner liaison [6] and subsequent audit research [4] found that up to 44% of patients with OA of the hip or knee referred for orthopaedic specialist consultation were unable to be offered an appointment, and were instead referred straight back to the referring GP without review or advice regarding ongoing management.

The Joint Clinic, a clinical service of the Orthopaedic Outpatient Department, Dunedin Hospital, was proposed and introduced to address the unmet need for secondary care consultation for people with late-stage hip and knee OA. Contemporary clinical practice guidelines for the management of OA recommend non-operative interventions – including exercise therapy and education – as core, first line management for all patients with hip or knee OA [7–9]. The Joint Clinic proposal was based on locally conducted research into the effectiveness of non-operative interventions [10–13]. The Joint Clinic was designed to contribute to the New Zealand Ministry of Health objective to provide *better, sooner, more convenient care* by improving the management of hip or knee OA at the interface between primary and secondary care [13, 14]. There is evidence to show that multidisciplinary collaboration and communication are essential to promote continuous, co-ordinated, patient-centred care with regard to OA [15].

The goal of this study was to conduct a comprehensive, longitudinal programme evaluation of the implementation of the Joint Clinic initiative. This programme evaluation was planned a priori and completed to assess whether the initiation and operation of the Joint Clinic achieved its four main objectives. These four objectives were to establish whether or not: 1) a physiotherapist-led clinic in a secondary care setting would be feasible as a method of meeting an unmet need for secondary care consultations and management in patients with hip or knee OA; 2) this new programme could be successfully implemented as proposed; 3) the new programme would operate as planned and be well accepted by stakeholders; and 4) the Joint Clinic was perceived to bridge the gap in care of those with OA of the hip and knee in a secondary setting in a cost-effective way, increase efficiency in its secondary care setting, and provide support for GPs in primary care.

## Methods

### The 'Joint Clinic' programme

**Fig. 1:**
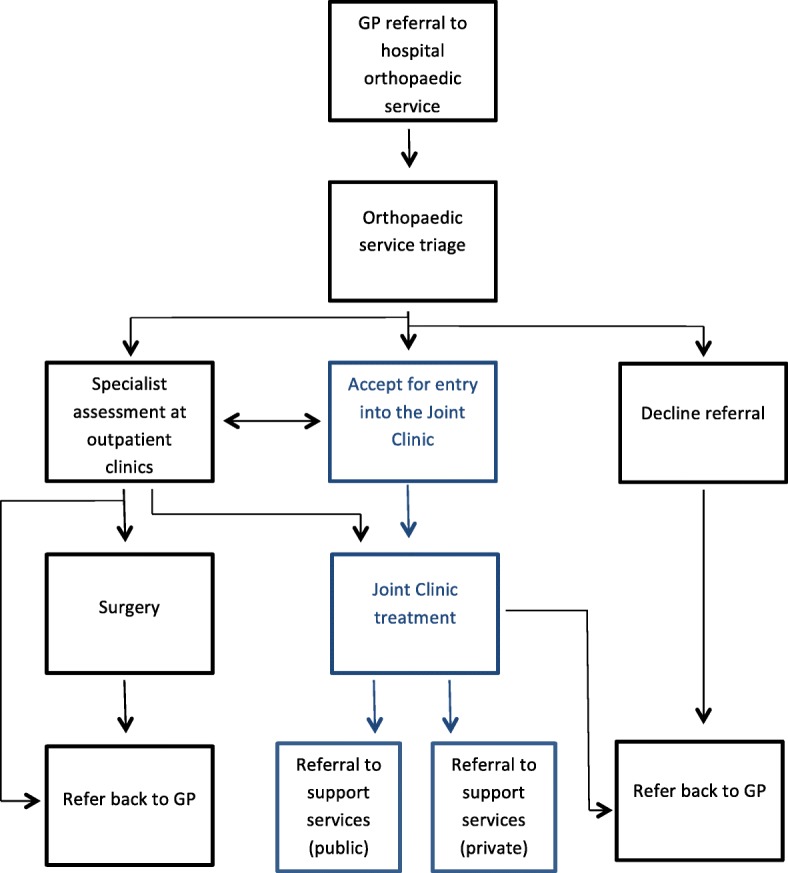
Referral pathway for OA patients referred from primary care to the Orthopaedic Department. Note: GP = general medical (family practice) practitioner

The Joint Clinic was developed as a clinical service of the Orthopaedic Outpatient Department at Dunedin Hospital. The patient referral pathway for OA patients referred from primary care to the Orthopaedic Department is illustrated in Fig. 1. To be eligible for inclusion, patients must have undergone clinical assessment by their GP and referred for orthopaedic consultation in secondary care (Dunedin Hospital) including current plain radiographs.

It was proposed that advanced competency physiotherapists would examine patients with hip or knee OA referred to the orthopaedic department and provide initial conservative management, education, referral and reassurance. A key component was referral to outpatient physiotherapy for a programme of exercise physiotherapy, when indicated, delivered either individually or in groups, in 6 visits of 40 min duration (see Additional file 1). Referrals could be made to an orthopaedic consultant, orthotics, dietetics, or community physical activity providers. All eligible patients managed in the Joint Clinic services would be followed up in clinic every 6 months until discharged. Discharge would occur when the programme course was completed, the patient stable, or when referral elsewhere was indicated. It was planned that the Joint Clinic would accomplish this with the support of an experienced orthopaedic nurse, consultant orthopaedic surgeons, and the Outpatient Physiotherapy Department.

The goals of the Joint Clinic programme were to increase efficiency in secondary care by decreasing time spent by Orthopaedic Consultants on patients not requiring surgery; to provide a much needed support for GPs in primary care by providing review and advice regarding ongoing management; to meet the unmet need described above; to improve patient outcomes; and demonstrate potential to make savings in both direct and indirect economic costs.

### Programme evaluation study design

**Fig. 2:**
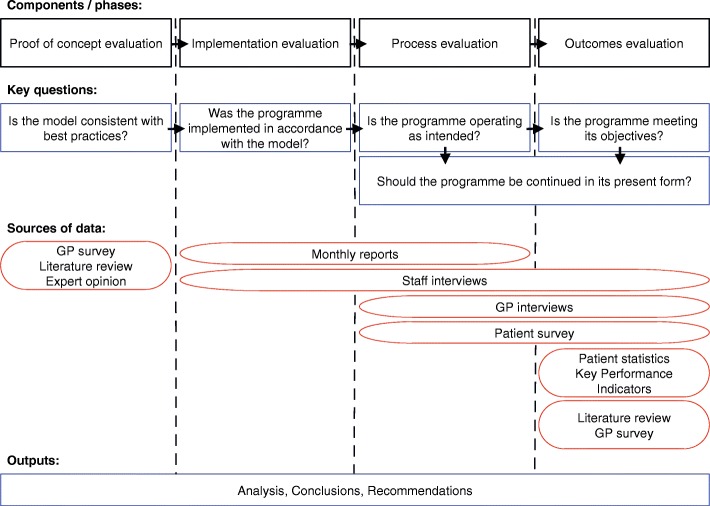
Components of the programme evaluation. Note: GP = general medical (family practice) practitioner

This study was an utilisation-focussed, end-to-end programme evaluation of the Joint Clinic. We structured and conducted the evaluation using the framework described by Hollander et al. [16] An overview of the evaluation structure and data collection methods are summarised in Fig. 2. The programme logic model is reported in Additional file 2. The initial phase was a *proof-of-concept evaluation.* In this phase the rationale for the programme was evaluated, the need for the service in the local community was assessed, and the key characteristics of the model were weighed against best practices in the field. An *implementation evaluation* was conducted, in an interim and a final phase. This assessed the extent to which the programme was executed in accordance with the proposed model. In concert, a *process evaluation* was done to assess whether the programme operated smoothly and efficiently, was adequately resourced and staffed, and was functioning as intended. Finally, an *outcomes evaluation* investigated whether or not the programme was achieving intended outcomes and objectives.

The primary outcome that the programme intended to address was unmet need for orthopaedic consultations for patients referred with OA, measured by number (proportion) of referrals sent back to the GP without consultation. Secondary outcomes included GP satisfaction with the service, acceptability of the programme by providers and patients, and service-level efficiency outcomes. Key outcomes assessed in each of the phases of the programme evaluation are summarised in the programme logic model (Additional file 2).

#### Literature review and expert opinion

The literature review and appraisal of expert opinion were conducted to indicate whether or not a physiotherapist-led clinic in a secondary care setting would likely be feasible and effective as a method of meeting an unmet need. The model was assessed against best practices, as identified by a review of the literature [15, 17–20], and by the leaders and staff of comparable OA clinics in Australia. Principals and staff from these clinics were consulted, site visits were conducted, and key characteristics of those services considered in the context of best practice recommendations [15, 17, 19, 20]. The programme was based on principles of chronic care [21–23]. Interventions included within the model, in particular the key physiotherapy component were investigated for support by clinical practice guidelines of effectiveness research [7–10], as well as a systematic review of cost-effectiveness [24].

#### Surveys of GPs, staff, and patients

Both pre- and post-implementation surveys and interviews were conducted, to assess objectives 2 and 3 relating to implementation and process. Survey design and delivery was based on best-practice evidence from the literature [25]. The surveys [see the appendices in Additional file 3] were intended to gauge Dunedin GPs’ satisfaction with the Orthopaedic Outpatient Department service, as well as their opinion regarding the need for the proposed new service. The pre-implementation survey consisted of three questions regarding access to an orthopaedic first specialist appointment (FSA). The post-implementation version consisted of eight questions; the first three questions were the same as those in the first survey, and the next five questions were about perceptions of the Joint Clinic operations. Participants were also invited to add free-text comments at the end of the survey.

All Dunedin GPs were mailed the survey, a reply-paid envelope and a pen [25]. After 4 weeks, non-responders with a known email address were sent an email with a link to the survey online. Non-responders without a known email address were posted a reminder letter, a second copy of the survey and a reply-paid envelope.

Each patient who had been seen for at least one follow-up appointment at the Joint Clinic by the end of year 1, was mailed a user perceptions survey [see the appendices in Additional file 3]. Eligible patients were contacted in the same manner as the GPs. Two weeks later non-responders were sent a reminder letter and another copy of the survey. The survey aimed to assess patient satisfaction at the Joint Clinic. The survey included questions about their satisfaction with wait time, the knowledge and expertise of staff, the treatment offered their overall experience, and whether or not they would recommend the Joint Clinic to others.

#### Interviews of GPs and staff

One-on-one interviews were conducted at the interim and post-implementation phases. The sampling frame included staff members from the Joint Clinic and the wider orthopaedic service, administration and management personnel, and GPs. In the interim evaluation, key stakeholders of the Joint Clinic were identified and invited to interview, and a chain sampling technique was used to recruit further interviewees. Two interviewers conducted the interim evaluation interviews. Chain sampling is a respondent-driven process, and involves identifying potential participants from key informants, and thus produces a ‘snowball’ effect [26]. In the post-implementation evaluation, six Southern District Health Board (SDHB) staff and seven General Practitioners (GPs) were invited to take part in a one-on-one in-depth interview. One interviewer (HH), familiar with the institution and environment and experienced in qualitative research, conducted the interviews. SDHB staff invited to take part were those identified as being closely involved with the Joint Clinic. GPs were selected, from those GPs referring patients that had had a follow-up appointment at the Joint Clinic, using a semi-random process to ensure that each GP interviewed was from a different practice.

The semi-structured interview questions aimed to assess the appropriateness, efficiency and effectiveness of the model’s implementation. Questions focussed on appropriate care provision, continuity of care, and competence of personnel [see the appendices in Additional file 3]. Interviews included open-ended questions to elicit large amounts of information from a relatively small number of key informants, to maximize data saturation. Thus, interviewees could produce specific answers as well as varied broad perspectives of individual experiences, opinions and motivations [27].

#### Monthly reports and patient-level data

To complement the surveys and interviews, monthly reporting on service-level and patient-level statistics were used to inform the outcomes evaluation. Monthly reports generated by the SDHB implementation project team provided statistics regarding department referrals, patient visits and pathways of care. A financial report was produced by the SDHB Business Analyst, and compared against the project budget.

### Data analysis

Survey data were analysed using Excel 2011 (Microsoft), and descriptive statistics were used to describe survey results. Themes were analysed from free-text comment data, and the main ideas were summarised.

All interviews were digitally recorded and transcribed by an independent transcription company. Transcriptions were checked against the interview recordings by the interviewer and corrected if necessary. Thematic analysis was carried out, which involved stages of familiarisation, identification of a thematic framework, indexing, charting and mapping and interpretation, based on the Framework Method [28]. NVivo software, version 10 (QSR International Pty Ltd), was used to organise the data [29].

Descriptive statistics were tabulated for service-level outcomes, the net marginal unit cost for all Joint Clinic services and physiotherapy treatments provided was calculated, and costs of programme implementation assessed against the budget allocated. Patient-level outcomes have been reported separately [30, 31].

## Results

### Surveys of GPs, staff, and patients

Pre-implementation surveys were sent to 111 GPs. Eighty-one respondents completed the survey, for a 73% response rate. The survey found that approximately 90% of GPs were ‘unsatisfied’ or ‘very unsatisfied’ with the access to FSA for their patients with advanced hip or knee OA. Once referred patients were seen, however, approximately 65% of GPs were ‘satisfied’ or ‘very satisfied’ with overall patient management provided, although approximately 30–35% expressed dissatisfaction with the overall management of their patients. Specific comments indicated that GPs thought *“getting patients into the system is difficult”* and *“too many referrals are returned unseen”,* and that *“Re-referral wastes time (GP and Specialist)”* [see the appendices in Additional file 3].

Post-implementation surveys were sent to 111 GPs. Fifty-eight surveys were completed, for a response rate of 52%. Most GP respondents (78%) had patients seen at the Joint Clinic. The majority of GPs (91%) remained ‘very unsatisfied’ or ‘unsatisfied’ with patient access to a FSA. Sixty percent of GPs reported being ‘satisfied’ or ‘very satisfied’ with overall patient access to the Joint Clinic; however, 40% reported being ‘unsatisfied’. Most GPs (91%) were ‘satisfied’ or ‘very satisfied’ with the quality and timeliness of feedback from the Joint Clinic appointment, and 76% were ‘satisfied’ or ‘very satisfied’ with the overall patient management regarding the Joint Clinic [see the appendices in Additional file 3 for figures and additional data].

Specific comments about the Joint Clinic indicated that GPs were “*… very pleased to have the Joint Clinic in the current environment where specialist appointments are so difficult to get”* and “*I think the joint clinic overall does a good job. I think patients also appreciate this service”.* However, some thought the Clinic added to the waiting problem, saying *“In my experience the Joint Clinic whilst no doubt well-intentioned functions as a further delay for patients whose need for joint replacement is already pressing by the time I have made a referral to orthopaedics”,* and suggested that *“…The joint clinic would be good for those at an earlier stage of the disease process - not those really for an operation but declined because of insufficient funding”.*

The patient survey indicated the majority of patients were ‘satisfied’ or ‘very satisfied’ with the knowledge and expertise of Joint Clinic staff (98%), the treatment plan given by Joint Clinic staff (89%), their treatment at Physiotherapy Outpatients (92%) and other treatments provided (82%). Most patients were ‘satisfied’ or ‘very satisfied’ to be seen by Joint Clinic staff rather than an Orthopaedic Surgeon (70%). The majority of patients (86%) were ‘satisfied’ or ‘very satisfied’ with the time they waited to be seen at the Clinic.

### Interim interviews of GPs and staff

Interim evaluation interviews were conducted among staff and GPs. After three phases of the chain sampling process, there were a total of 21 potential respondents, of which 16 were interviewed. These comprised six Orthopaedic Department or Joint Clinic clinicians, one allied health clinician, seven hospital administrative or managerial staff, one SDHB Māori (New Zealand’s indigenous peoples) liaison, and one GP. Overall, data from the interim implementation evaluation indicated that the Joint Clinic had been implemented in close concordance with the proposed model and was well accepted by the key stakeholders, staff, and patients. Six major themes resulted: staffing, appropriate care provision, care coordination, promotion of the service, the Joint Clinic model and Hauora Māori (health and wellbeing of Māori).

Recurrent themes relating to staffing included high levels of confidence in the competence of personnel, and concerns regarding adequacy of allocated administrative staff time in light of heavier than expected additional workload. One aspect of the proposed model that was not implemented was the employment of “advanced physiotherapy practitioners”. Instead, due to loss of the initial lead physiotherapist the Joint Clinic role was filled by an experienced physiotherapist without advanced practice experience or specific OA expertise. However a training programme had been provided. Staff surveys found that adequate leave cover for both the physiotherapist and the nurse were lacking. A physiotherapist was allocated and trained for ‘back-up’ cover, but became unavailable.

Some planned aspects were not concordant. It was found that some GPs wrote referrals of patients directly to the Joint Clinic, instead of following the existing protocol that referrals should be triaged by the orthopaedic surgeons, as any other referral would be. Also, clinic staff reported occasional difficulty in accessing orthopaedic surgeons for discussion regarding complex patients, leading to gaps in communication. The lead orthopaedic surgeon’s time spent discussing cases with Joint Clinic staff had not been budgeted a priori.

### Final implementation interviews

In the final implementation evaluation, six SDHB staff and seven GPs were invited to take part in one-on-one in-depth post-implementation interviews; all but one GP accepted and were interviewed. Six themes resulted from the data: clinic impacts, clinic value, access, knowledge and understanding of the clinic, communication, and the future of the clinic.

The main impacts of the Joint Clinic were generally seen as positive, as patients who previously would have been returned to their GPs were being seen at a secondary level. Providers commented that *“...it’s absolutely plugged a huge gap...”* (SDHB staff)*, “…instead of the referrals being triaged and sent back to the GP, not being seen at all... they’re now being seen”* (SDHB staff) *“…more quickly, more efficiently, and more to the point...and help GP[s] to, to manage a long term problem”* (GP).

Interviewees had the impression that patients valued the service as well, and had benefited, at least psychologically, commenting that *“...patients do have the perception that they, that something’s happening”* (GP)*, and “All of them [patients] have had an improvement in their function. That doesn’t translate into leading, needing less pain relief. It doesn’t translate into not needing joint replacement. It does translate into believing that they haven’t been abandoned by the system, into realising that they will recover from what is major surgery and holds considerable fear for most people still”* (GP).

The perception was raised that some may patients express initial disappointment because they didn’t get to see an orthopaedic surgeon: *“...patients might feel fobbed off if the purpose of the Joint Clinic has not been explained to them”* (GP)*; “There are some patients that are initially quite upset or potentially frustrated with actually the fact that they’re not seeing an orthopaedic doctor. However, I think with just a little bit of explanation of what that clinic actually involves, I think they realise that what the clinic has to offer is really, is really quite beneficial for them”* (SDHB).

The Joint Clinic was valued by the GPs interviewed, but the idea was raised that not all patients would gain substantial value from the clinic. While typical GP comments conveyed that they *“…think it’s enormously valuable”* (GP), and *“Most of my patients would be enormously grateful for the care they receive. All of them have had an improvement in function”* (GP), some also commented that *“They like meeting the people, but it hasn’t helped their hip”* (GP).

The SDHB staff interviewed generally agreed the programme was helping to meet unmet need, and there was good acceptance of the programme among the interdisciplinary team*. “It’s helping the demand for FSA which it was, is also in excess of what we could supply”* (SDHB staff) and *“...the GPs are definitely coming on board too. Because, I mean on their referrals they’re actually, quite a few of them are very proactive in writing that they think their patient would be suitable for the Joint Clinic”* (SDHB). The consensus was unequivocal that *“the allied health team do a really great job with it”* (Participant 2, SDHB) and*“There’s a lot of trust and respect there within that relationship [between staff members]”* (SDHB).

Lack of clarity and understanding about the Joint Clinic was a noted weakness: *“I think the perceptions of what the Joint Clinic’s trying to achieve or is actually doing differ across the primary care, secondary care sort of interface. So I’m not sure it’s, people are totally clear about what’s happening”* (SDHB). During interviews it was suggested that, to be successful in the future, the Joint Clinic needed to increase its visibility, communicate its mission clearly to stakeholders, maintain its funding, and decrease attrition among physiotherapists and staff. Further details of the themes, subthemes, and additional data are available in the online-only supplemental material [see the appendices in Additional file 3].

### Service level outcomes

Over 2 years, 358 new patients and 279 follow-ups were seen at the Joint Clinic, for a total of 637 patient visits during 2 years of operation (Table 1). Un-notified ‘did not attends’ (DNAs) were low with only 11 DNAs overall (3.8%) in the first year, and 16 DNAs (4.3%) in the second year.

**Table 1:** Description of the patients and patient pathways of the first 2 years of Joint Clinic operation

	Total	
Patients referred to Joint clinic	376	
Declined	9	2.4%
Did not attend	9	2.4%
Patients attending Joint Clinic	358	
Patient characteristics		(of 358)
Age (SD)	76	9.8
Female	200	55.9%
Hip OA^a^	155	43.3%
Knee OA^a^	199	55.6%
Not OA^a^	19	5.3%
Met inclusion criteria^b^	339	94.7%
Joint Clinic management		
Initial consultation	358	95.2% (of 376)
1 follow-up	252	74.3% (of 339)
2 follow-ups	114	36.6% (of 339)
3 follow-ups	28	8.3% (of 339)
mean (SD) visits	2.1	0.91
Referred for FSA:		
Initial visit	59	16.5% (of 358)
Subsequent visit	74	
By another service	15	
Total	148	41.3%

*GP* General medical practitioner (family practice physician). ^a^OA as the primary cause of hip or knee symptoms was the inclusion criterion for Joint Clinic management; sums to > 100 due to multisite OA. ^b^OA of the hip or knee

The primary outcome of reducing unmet need for secondary care consultations and management in patients with hip or knee OA was achieved, with the proportion of GP referrals for hip or knee OA returned without offer of consultation reduced by 90%. Increased efficiency in its secondary care setting was demonstrated by reductions in overall (all-cause) referrals returned to GPs without consultation, despite an overall decrease in FSAs provided by the Department. The Joint Clinic resulted in an overall 5.7% increased capacity of the Orthopaedic Outpatient service to provide initial consultations compared with the year prior to implementation of the Joint Clinic. These changes were observed on a background of a decreased volume of referrals received overall (Table 2).

**Table 2:** Reductions in the number of patient referrals received by Orthopaedic Outpatients, number of First Specialist Assessments (FSAs) delivered, and number of referrals sent back to the GP without consultation: baseline and first 2 years of Joint Clinic operation

	Year 0^a^	Year 1	Change Year 0–1	Year 2 (cumulative total)	Change Year 0–2^b^
Referrals	2,078	1,584	−24%	1539 (3123)	−25%
FSAs	1,305	1,134	−13%	1267 (2401)	−8%
Referrals returned to GP	557	390	−30%	462 (852)	−24%
Referrals returned to GP [hip, knee OA only]	74	5	−93%	10 (15)	−90%

^a^the year prior to Joint Clinic implementation; ^b^annual change = 1-([cumulative total/2]/year 0 total); *GP* General medical practitioner (family practice physician)

Patient level outcomes have been reported elsewhere [30, 31]. In summary, approximately 60% of patients were managed non-operatively by the Joint Clinic, with a significant improvement (18% improvement on baseline Oxford score, *p* = .0013 for change by paired, 2-tailed t-test) noted in that group; the remaining 143/358 (40%) were referred for FSA, with 115 (80%) received or were listed for surgery [31]. At referral to Joint Clinic, no differences in age, sex, or patient-reported outcome measures were evident between those with hip versus knee OA, however mean BMI was higher in the knee OA group. Patients with knee OA improved significantly, on average, while patients with hip OA were more likely to deteriorate significantly and require surgery [30].

### Cost-effectiveness

The net marginal unit cost for all Joint Clinic services and physiotherapy treatments provided in the Physiotherapy Outpatient Department decreased in each financial year from $550 per patient in year one to $384 per patient in the second year of operation, due to greater efficiency of clinician time allocated. The Joint Clinic operated significantly below budget in each financial year due to lower than budgeted total personnel costs.

## Discussion

As the world’s population ages, health care systems will come under greater pressure to meet the increasing burden of all musculoskeletal disorders, and OA in particular [1]. In New Zealand, the demand for joint replacement surgery is predicted to rise dramatically, placing substantial pressure on orthopaedic outpatient consultation services, which assess potential candidates for joint replacement surgery, and manage end-stage OA [3]. The results of this programme evaluation of an end-stage hip and knee OA Joint Clinic demonstrates that a service dedicated to meeting the unmet need in this area can be successfully implemented at the interface of primary and secondary care.

The proof-of-concept model for the Joint Clinic was supported by best-practice literature for OA care and by external experts [15, 17–20]. The Joint Clinic service delivery model was similar to others, such as those presented in the UK National Health and Australian healthcare systems [17, 32], and was founded on clinical evidence and experience from the Management of Osteoarthritis (MOA) Research Trial programme conducted locally at the University of Otago [10]. The MOA Trial was a randomised clinical trial which included an economic evaluation [10–12]. This local evidence was supported by broader evidence for both effectiveness [10] and cost-effectiveness [11, 12]. The Joint Clinic structure also included several elements that are consistent with the Wagner Chronic Care Model, a model which aims to support patients with chronic conditions to self-manage their condition [21–23, 33, 34].

Government health policy [35], workforce recommendations [36], and local need [5] supported the rationale for the programme. The primary outcome of the Joint Clinic was intended to be reduction in unmet need for primary care referrals to secondary care. In Dunedin, the local unmet need is centred around access to orthopaedic FSAs and wait times for surgery, both of governed by the rationale for resource allocation [37]. This primary outcome was reduced by 90%. We have established that the new programme was successfully implemented as proposed, with the exception of the inability to retain the employment of “advanced physiotherapy practitioners”. However the use of an experienced physiotherapist after provision of a training programme was successful and stakeholder satisfaction with the clinical staff was very high. We also were able to establish that the new programme was able to operate as planned and be well accepted by stakeholders. Dissatisfaction with access to orthopaedic surgeon FSAs was unchanged, post-implementation, from the high level (90%) reported pre-implemention, despite Joint Clinic facilitating access to FSA for 40% of patients who would otherwise have been sent back to the GP without consultation or advice. The qualitative data of the free-text responses support the interpretation that this reflects ongoing frustration with orthopaedic secondary care access problems more generally. Those data also indicated that the Joint Clinic was a helpful alternative, with some concerns also expressed that it was merely a ‘delaying tactic’ stalling access for patients who really required surgery/FSA.

The data indicated that the Joint Clinic was perceived to bridge the gap in care of those with OA of the hip and knee in a secondary setting satisfactorily, and provided welcome support for GPs in primary care. Referral volumes were lower than anticipated during implementation, and then increased to the intended capacity. The establishment of the Joint Clinic was observed to increase efficiency of orthopaedic surgeon appointment resources in the secondary care setting, in terms of increased provision of patient assessments overall, and shifting ‘non-surgical’ consultations from orthopaedic surgeons to Joint Clinic. The unit cost was lower than many other unit costs for non-pharmacological, non-surgical interventions for osteoarthritis reported in the literature, which indicate that the cost of intervention being more than recouped by savings in other health services over 1–2 year [12, 24, 38] . The SDHB concluded the Joint Clinic was a cost-effective use of resources and renewed programme funding. The service concluded the Joint Clinic was a cost-effective use of resources, and resolved to continue the new programme indefinitely.

Limitations of the evaluation include the uncertainty that results from background changes to referral patterns and Department capacity unrelated to the implementation of the programme. Overall referrals to the Department for FSA decreased in year 1, and recovered somewhat in year 2 but not to the level observed pre-implementation. The reason for this decrease cannot be concluded from the evaluation data, but may be due to ongoing education of GPs by the Orthopaedic Department on appropriate referral criteria and prioritisation criteria. We also cannot draw conclusions regarding the generalisability of the Joint Clinic to other regions or services with differing referral drivers, unmet need, or policy mechanisms.

Stakeholder interviews and survey data raised the concept of a primary care version of a Joint Clinic-like service, targeting OA earlier in the course of disease. The case for translating this service to a primary care setting is strong, to target OA earlier in the course of the disease. This approach is supported by research evidence [15, 17], indicating that conservative care is more effective in patients at earlier stages of OA progression [39, 40], and that early intervention can delay or prevent the need for joint replacement surgery [41, 42].

## Conclusions

This programme evaluation has established that a physiotherapist-led clinic in a secondary care setting is feasible, effective in reducing unmet need, and is acceptable to all stakeholders. The Joint Clinic offers another option for patients with OA of the hip and knee, and the services that provide care in a secondary setting. The service appears to provide a much-needed support for GPs in primary care. Thus the Joint Clinic services appear to be sustainable and there is the capacity for increased volume to extend the scope of the service.

## Additional files

Additional file 1: Guidelines for the delivery of the outpatient physiotherapy intervention: The Joint Clinic (PDF 69 kb)

Additional file 2: The Joint Clinic programme logic model (PDF 59 kb)

Additional file 3: Appendices to the Methods and Results (PDF 571 kb)

## Data Availability

The data used and/or analysed during the current study are included in this published article and its supplementary information files [Additional file 3], and is also available from the corresponding author on reasonable request.

## Abbreviations

CPAC: Clinical Priority Assessment Criteria
FSA: First Specialist Assessment
GP: General Practitioner
OA: Osteoarthritis
QALY: Quality Adjusted Life Year
SDHB: Southern District Health Board
SMO: Senior Medical Officer
