# Supplementary material for: Implementation of a ‘Joint Clinic’ to resolve unmet need for orthopaedic services in patients with hip and knee osteoarthritis: a program evaluation
Source: BMC Musculoskelet Disord. 2019 Jul 12;20:324. doi: 10.1186/s12891-019-2702-1 (PMC6624903; doi:10.1186/s12891-019-2702-1)
Supplement: Supplementary file 1 — Guidelines for the delivery of the outpatient physiotherapy intervention: The Joint Clinic (PDF 69 kb) [file 12891_2019_2702_MOESM1_ESM.pdf]

ADDITIONAL FILE 1:

GUIDELINES FOR THE DELIVERY OF THE OUTPATIENT PHYSIOTHERAPY INTERVENTION: THE JOINT CLINIC

GENERAL PRINCIPLES

The principles for treating Joint Clinic patients is as easy as 1-2-3-4

1: Each patient is an individual: assess their impairments and tailor the programme to match the needs of the individual, and challenge their current level of ability. It's not a recipe book: each patient must get individualized care.

2: There are two initial physiotherapy treatment options: exercise therapy and manual therapy. You should focus on only **one at a time**. Everyone must get exercise therapy.

a) **The goal of exercise therapy is to create *physiological change*, i.e. you must deliver a sufficient dose of resistance/duration difficulty to challenge the neuromuscular and cardiorespiratory systems.** Everyone must get exercise therapy, however if your examination finds impairments that would benefit from manual therapy, devote the first several sessions to manual therapy only.

b) **The goal of manual therapy is to employ *tissue altering* techniques, i.e. you have to go into resistance.** Treat each impairment with manual techniques that will take the joint well into the end of its available range at least once in a treatment session. Use the highest dose of mobilization appropriate: i.e. at least one set of grade III, IV, or V each session. Once good progress has been made over several sessions (including prescription of range-of-motion reinforcing activities for the patient to do regularly at home), exercise therapy should be introduced. Some overlap is acceptable.

3: Adhering to the following **three very important principles** will ensure delivery of an effective programme of therapy:

1. **Fidelity** – that the treatment delivered stays 'true' to what was shown to be effective in the MOA Trial [1, 2].
2. **Dose** – that each participant gets a 40 minute treatment session that is sufficiently challenging to stimulate physiological change
3. **Progression** – that each participant is started at a high enough level, and is progressed to higher levels of resistance, stretch, repetitions, difficulty and/or duration, such that the programme remains challenging and continues to progressively stimulate physiological change.

4: The exercise therapy protocol has four components – and everyone gets exercise therapy. The exercise therapy protocol is based on the protocol shown to

be effective in the MOA Trial [1, 2]. It is a multi-modal exercise protocol consisting of four components:

1. Aerobic/warm-up;
2. Strengthening;
3. Stretching;
4. Neuromuscular control

EACH PARTICIPANT PERFORMS ALL FOUR COMPONENTS OF THE PROGRAM

Aerobic. To be performed for up to 10 minutes at moderate level as warm-up. This does not have to be under your direct supervision.

Strengthening. Begin with a dose of 3 sets of 10 repetitions, at a level of resistance appropriate to the individual's ability, with a 3 second hold. Adjust up (or down if necessary) from there, based on response to treatment.

Stretching. If impairments requiring stretches are found, effective stretches must be performed each session until goals are met.

Dose: 1 minute total, using 20-60 second hold times

Neuromuscular control. Choose from the range of exercises provided in the programme. Select the most challenging exercises the patient can achieve safely.

Dose: 2 minutes each exercise. The aim is 6 minutes of exercise in this category. You may select 3 different exercises, or repeat any exercise more than once.

The exercise therapy and manual therapy intervention protocols [3] are available at:

https://ourarchive.otago.ac.nz/handle/10523/7354

References:

1. Abbott JH, Chapple C, Pinto D, Wright AA, Leon de la Barra S, Baxter GD et al. Exercise therapy, manual therapy, or both, for management of osteoarthritis of the hip or knee: 2-year follow-up of a randomized clinical trial. Osteoarthritis Cartilage. 2014;22(Supplement):S51.
2. Abbott JH, Robertson MC, Chapple C, Pinto D, Wright AA, Leon de la Barra S et al. Manual therapy, exercise therapy, or both, in addition to usual care, for osteoarthritis of the hip or knee: a randomized controlled trial. 1: clinical effectiveness. Osteoarthritis and Cartilage. 2013;21(4):525-34. doi:10.1016/j.joca.2012.12.014.
3. Abbott JH. Management of Osteoarthritis: A guide to non-surgical intervention. Dunedin, New Zealand: The Clarity Group; 2014. ISBN: 978-0-473-28968. Available at: https://ourarchive.otago.ac.nz/handle/10523/7354
