# Supplementary material for: Implementation of a ‘Joint Clinic’ to resolve unmet need for orthopaedic services in patients with hip and knee osteoarthritis: a program evaluation
Source: BMC Musculoskelet Disord. 2019 Jul 12;20:324. doi: 10.1186/s12891-019-2702-1 (PMC6624903; doi:10.1186/s12891-019-2702-1)
Supplement: Supplementary file 2 — The Joint Clinic programme logic model (PDF 59 kb) [file 12891_2019_2702_MOESM2_ESM.pdf]

Additional File 2: The Joint Clinic programme logic model

Definition of the problem	Inputs		Outputs<br>Implementation →		Outcomes		
	**Components**	**Activities**	**Activities**	**Target**	**Short term**	**Intermediate**	**Long term**
Osteoarthritis (OA) principally affects the older population, and is the most common form of arthritis. The prevalence of OA increased 32% in the last decade.<br><br>In New Zealand, OA of the hip and knee is the most common condition for which joint replacements are indicated. As the population ages, this will place significant stress on the health resources in NZ.<br><br>In Dunedin, there is a disproportionately low number of orthopaedic specialists. Up to 44% of patients with OA of the hip or knee referred for orthopaedic consultation are unable to be offered a specialist consultation.<br><br>Evidence supports that multidisciplinary collaboration and communication are essential to promote continuous, co-ordinated, patient-centred care for people with OA.	Identify clinical need<br><br>Proposal of the 'Joint Clinic' as a solution to unmet need.<br><br>Grant from National Health Board (via SDHB)<br><br>Research conducted at Centre for Musculoskeletal Outcomes Research, University of Otago	Finalize model and plans<br><br>Develop key aims and evaluation plan<br><br>Develop clinical pathway<br><br>Develop staffing plan<br><br>Develop method of reporting<br><br>Develop method of liaising with GPs and Orthopaedic staff/surgeons	Complete monthly implementation and outcomes reports<br><br>Collaborate with SDHB, GPs, and Orthopaedic outpatient services<br><br>Coordinate te programme across relevant sectors	Share assessments with GPs and surgeons<br><br>Gain support from stakeholders and actors<br><br>Educate GPs about existence of Joint Clinic and its role in the patient pathway	Collaborative planning and goal setting<br><br>Achieve outcomes targets:<br>- primary outcome = reduce unmet need for orthopaedic consultations for patients with OA<br><br>Complete programme evaluation report for funders	Address issues raised by evaluation<br><br>Publish outcomes in peer-reviewed journals<br><br>Increase or meet capacity	Attain funding security for continuation of the service<br><br>Replicate Joint Clinic model with other musculoskeletal conditions

Time →

Notes: GP = general medical (family practice) practitioner; OA = osteoarthritis; SDHB = Southern District Health Board
