# Supplementary material for: Implementation of a ‘Joint Clinic’ to resolve unmet need for orthopaedic services in patients with hip and knee osteoarthritis: a program evaluation
Source: BMC Musculoskelet Disord. 2019 Jul 12;20:324. doi: 10.1186/s12891-019-2702-1 (PMC6624903; doi:10.1186/s12891-019-2702-1)
Supplement: Supplementary file 3 — Appendices to the Methods and Results (PDF 571 kb) [file 12891_2019_2702_MOESM3_ESM.pdf]

ADDITIONAL FILE 3:

Appendix 1. Dunedin GP Survey – July 2012

A pre-implementation survey of Dunedin GPs' satisfaction with the Orthopaedic Outpatient Department service at Dunedin Hospital. Conducted and reported by Dr Cathy Chapple under supervision of Dr Haxby Abbott..

This survey was carried out during March and April 2012 and referred to the previous 6-months experience. The survey was constructed using best-practice evidence from the literature on ways to improve responses to questionnaires. The plan is to repeat the survey as part of the one-year evaluation of the service.

The questions were:

How satisfied are you with:

1. The ease of access (including waiting time) for a first specialist appointment, for your patients with knee or hip OA?

Very **un**satisfied ☐	**Un**satisfied ☐	Satisfied ☐	Very satisfied ☐

2. The quality and timeliness of feedback from the appointment? (Clinic letter)

Very **un**satisfied ☐	**Un**satisfied ☐	Satisfied ☐	Very satisfied ☐

3. Overall patient management (including treatment plan/implementation, education/advice)?

Very **un**satisfied ☐	**Un**satisfied ☐	Satisfied ☐	Very satisfied ☐

Results:

- 111 Dunedin GPs on SDHB community contact list were sent the survey
- 81 responses = 73% response rate
- 74 responses collated/analysed (6 GPs do not see OA (student health/24hrs)/1 GP left Dunedin)

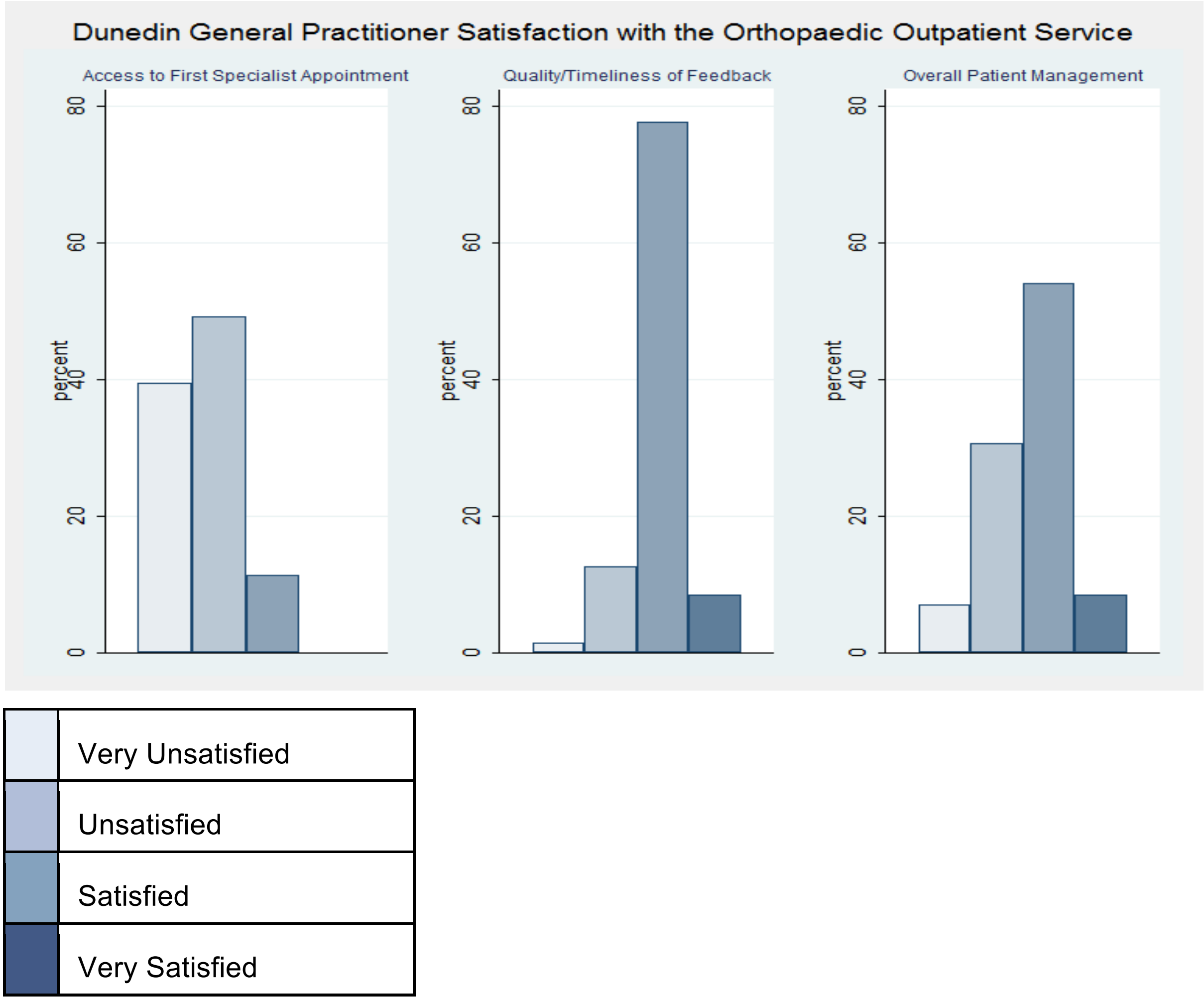

Examples of free text comments:

Getting first appointment (n=20)

- “Getting patients into the system is difficult”
- “Satisfied with care if they can get an appointment”
- “Too many referrals are returned unseen”
- “Re-referral wastes time (GP and Specialist)”

Points system for surgery (n=11)

- “patients unable to get enough points for surgery although quality of life affected by pain and limited movements”
- “The orthopaedic surgeon often considers surgery the most appropriate Rx but the patient gets bumped because of "points" system”

Appendix 2. Dunedin GP Survey – February 2014

A post-implementation survey of Dunedin GPs' satisfaction with the Orthopaedic Outpatient Department service at Southland Hospital. Conducted and reported by Dr Helen Harcombe under supervision of Dr Haxby Abbott..

Introduction

The aims of this survey were to: 1) measure General Practitioner (GP) satisfaction with the Southern District Health Board (SDHB) Orthopaedic Outpatient service for patients with hip or knee osteoarthritis, and 2) investigate GP views on the Joint Clinic service.

Methods

At the end of January 2014, all Dunedin GPs were sent the postal survey, a reply-paid envelope and a Management of Osteoarthritis (MOA) pen. After four weeks, non-responders with a known email address were sent an electronic version of the survey as well as a link to survey monkey. Those non-responders without a known email address were posted a reminder letter, a second copy of the survey and a reply-paid envelope. The survey consisted of eight questions. The first three questions were regarding access to a first specialist appointment (FSA) and were the same as those in the 2012 survey of Dunedin GPs. The following five questions were about the Joint Clinic and were new for this survey. Participants were also invited to add extra comments at the end of the survey. Data were analysed using Stata version 12 (StataCorp).

Results

Response rate

The survey was sent to 111 GPs. For several potential participants, the survey was returned to sender (n=2) or returned as it was not applicable to that GPs patient population (n=1). Of the 108 potential participants remaining, the survey was completed by 58 GPs giving a participation rate of 52%.

Findings regarding FSA

1. Most GPs (91%) were 'very unsatisfied' or 'unsatisfied' with access to FSA with an orthopaedic surgeon.

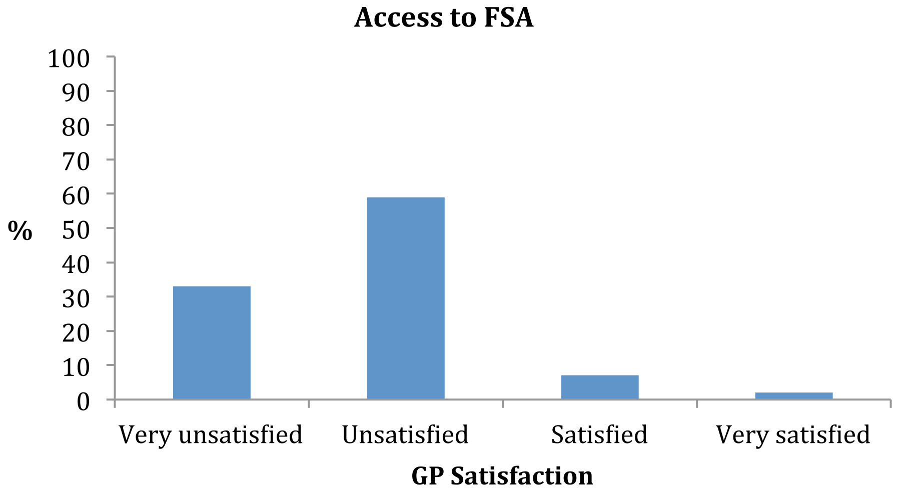

2. Most GPs (95%) were 'satisfied' or 'very satisfied' with the quality and timeliness of feedback from the appointment (clinic letter).

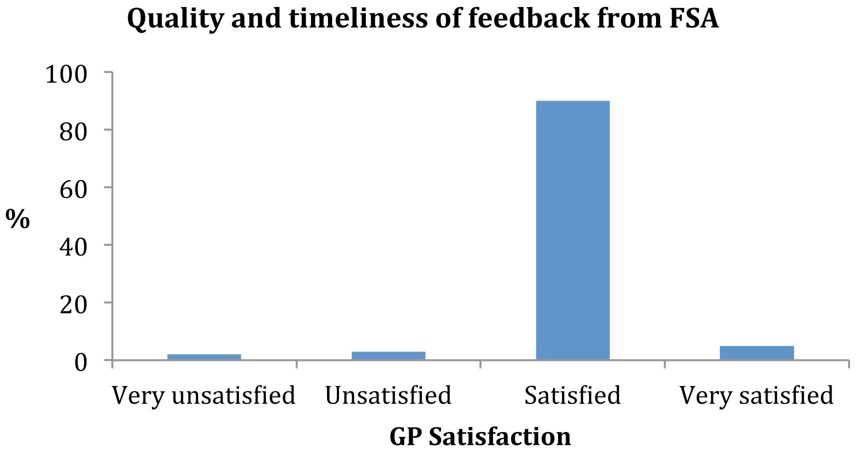

3. The majority of GPs (62%) were 'satisfied' or 'very satisfied' with the overall patient management with regard to FSA. However, 28% were 'unsatisfied' and 10% were 'very unsatisfied.'

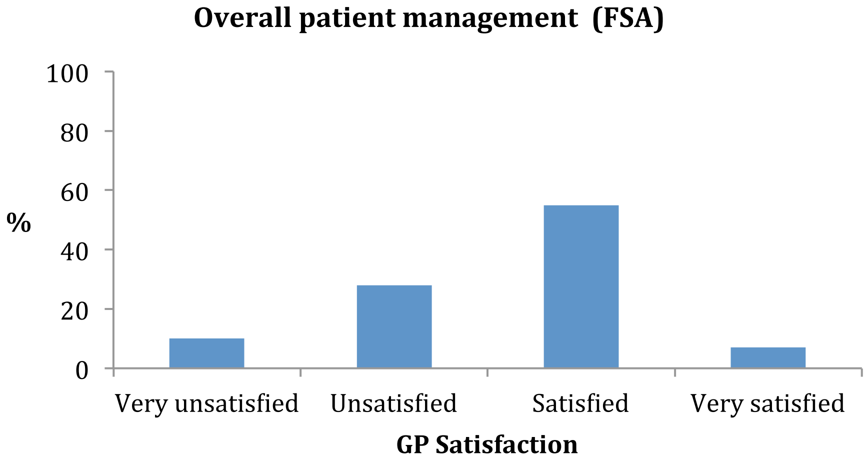

Findings regarding the Joint Clinic

4. Most GPs (78%) had patients that had been seen in the Joint Clinic. However, 9% (n=5) had no patients seen in the Joint Clinic and 14% (n=8) were unsure whether any of their patients had been seen by the Joint Clinic.

5. Of those GPs who had patients that had been seen in the Joint Clinic (n=45), 60% were ‘satisfied’ or ‘very satisfied’ with the access to the Joint Clinic.

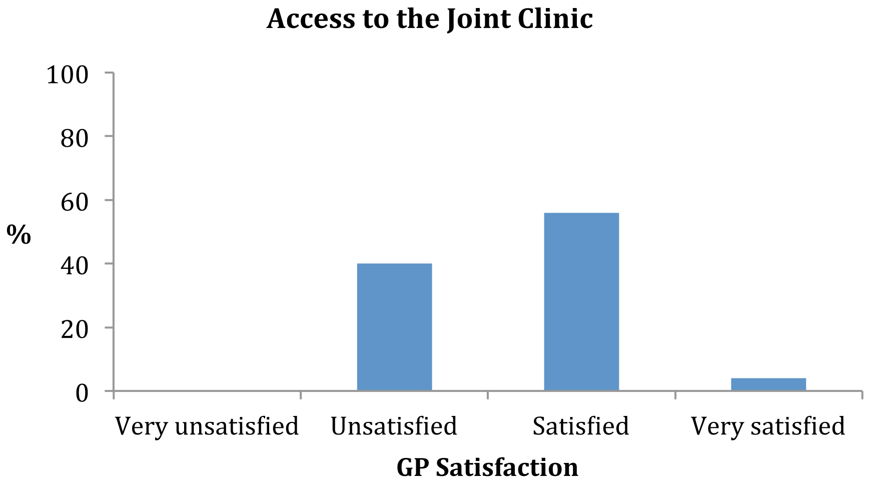

6. Most GPs (91%) were ‘satisfied’ or ‘very satisfied’ with the quality and timeliness of feedback from the Joint Clinic appointment.

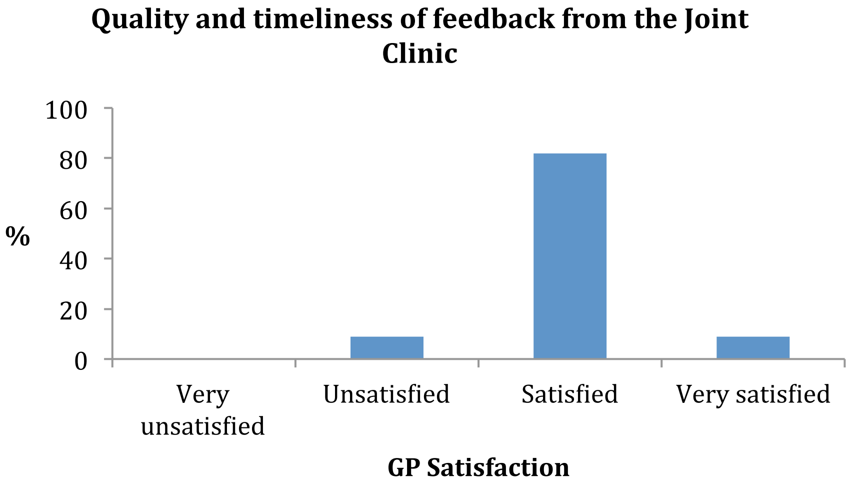

7. Most GPs (76%) were ‘satisfied’ or ‘very satisfied’ with the overall patient management regarding the Joint Clinic

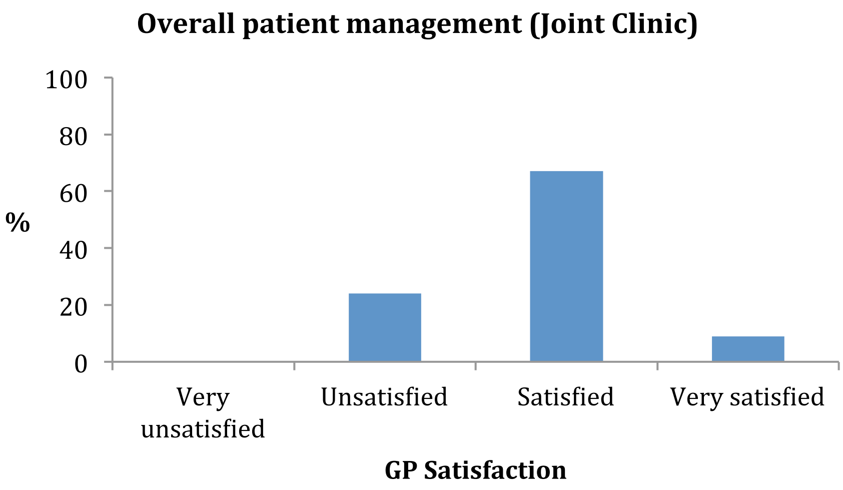

8. The majority (56%) of GPs who had patients that had been seen in the Joint Clinic were ‘very unsatisfied’ or ‘unsatisfied’ with access to (including waiting time for) recommended treatments such as physiotherapy/supervised exercise, weight loss dietary advice, education/information or surgical assessment. However, 36% were ‘satisfied’ and 4% were ‘very satisfied’ with access to recommended treatments.

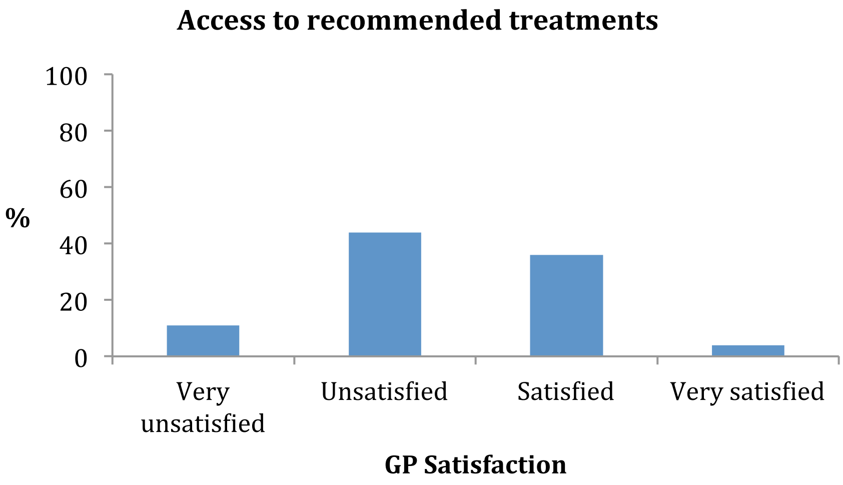

Missing data 4% (n=2)

Free text comments

Free text comments were provided by 25 GPs (43%). Some GPs commented on more than one aspect. Comments incorporated a variety of aspects including FSAs, availability of joint replacement surgery and aspects of the Joint Clinic.

Comments regarding FSA

Comments regarding FSA referred to a lengthy wait for appointments, delay in getting letters back from clinic appointments, comments about FSA management and an unacceptably long wait for, and difficulty getting, joint replacement surgery for their patients.

FSA wait

- "In general, waiting times for specialist review are very long for patients coming with severe pain daily"
- "Waiting doesn't seem to have changed"
- "Problem is the wait. We refer in the first place when the patient has had enough!"
- "Many of my referrals to orthopaedics are rejected which is frustrating as they take a long time to write! ... I realise this is probably due to not enough funding/surgeons but it would be really helpful if we could have some detailed guidelines of when to refer. We could also use more of these to show patients who are demanding referral. Good for other non OA orthopaedic problems too"
- "There is still a very long wait for patients needing assessment for hip and knee joint OA.
- "I'm sure you are trying hard but the end result in general practice is - many people wait too long to be assessed…"

FSA Letters

- "Letter from you [FSA] can take a long time to get through the typing pool - often > 1 month by which time we have seen the patient and don't have the new information. This is a general impression of ALL outpatient responses, not specifically the orthopaedic service."

FSA management

- "Poor advice on patient symptom management"
- "[Satisfactory] once achieved!"
- "I would appreciate some advice on non surgical management for those who do not qualify for surgery. Often they have reasons not to use NSAIDs and pain management is inadequate. Often the only outcome of the appt is simple yes-no to being eligible for surgery. Ongoing primary care management can be very challenging!!"

Surgery wait/capacity

- "High needs, incapacitated patients are waiting too long for surgery"
- "It is still highly irritating when deserving patients can't get their operations…."
- "Need more TK/THRS"
- "…many people who really need a joint replacement are not getting them."
- "My main problem is the difficulty in getting surgery for patients who would obviously benefit"
- "We need more surgery for joint replacements"
- "Patients have to be pretty disabled in Dunedin before they can get surgery"
- "The waiting list and points needed to be on this for surgery are really unacceptable"
- "I am distressed at the level of dysfunction and pain needed to get the points for surgery. 'Active Review' and 'GP management' does not help the patient"
- "Ongoing issue is that patients needing THR are put on 'review' list and deteriorate markedly and have to be referred back. The threshold for surgery is set too high and leads to too great disability before treatment is Rx is offered"

Comments about the Joint Clinic

Comments about the Joint Clinic were mixed with some suggesting it was a delay for patients and not targeted to the most appropriate group. However, others noted that patients had appreciated the Clinic and found it helpful.

Positive aspects

- "It is still highly irritating when deserving patients can't get their operations but the Joint Clinic is a really helpful compromise"
- "My patients have found the Joint Clinic very helpful but some will still [need] joint surgery"
- "Good for patients initiative"
- "Am very pleased to have the Joint Clinic in the current environment where specialist appointments are so difficult to get"
- "I think it is marvellous that you are offering physio for those not able to get on waiting lists as many of my OA patients cannot afford community physio without ACC - I had been encouraging them to pay for this and not referring them for ortho assessments at all…"
- "I think the joint clinic overall does a good job. I think patients also appreciate this service"
- "Good to have a useful alternative to seeing specialist - patient gets a chance for 2nd opinion which usually confirms my advice that not ready for joint replacement yet…(or can't be done in Public system)…has the mana of the specialist system"

Negative aspects

- "In my experience the Joint Clinic whilst no doubt well-intentioned functions as a further delay for patients whose need for joint replacement is already pressing by the time I have made a referral to orthopaedics"
- "To be honest this felt like a 'stalling' tactic"
- "Ver[y] small sample size, one, but patient perception was that the Joint Clinic was a delaying tactic to prevent access to an orthopaedic surgeon + joint replacement, which was clearly needed and subsequently happened"
- "The joint clinic is trying to help those who are 'past' their help + really need an operation. The joint clinic would be good for those at an earlier stage of the disease process - not those really for an operation but declined because of insufficient funding"
- "I am still getting patients who I feel should go straight to FSA who are being diverted to joint clinic. I wonder whether, with some education, the TRIAGE aspect of the joint clinic process would be better managed in primary care. Conversely there are patients that I am still reluctant to refer unless they will definitely go straight to joint clinic as I am quite sure an FSA is not warranted (and the DHB has discouraged us from making these referrals in the past)"

Overall patient management (Joint Clinic)

- "It would be useful if the physio could feed directly in to the orthopaedic clinic when the physio feels they are eligible for surgery. I have been asked to refer two patients for joint replacement which is unnecessary, time-consuming and creates further delays"

Other

- "The physio clinic is good"
- "Can we refer directly to the joint clinic?" [Unclear whether this is a question or a request]

Discussion

Overall, most GPs were 'very unsatisfied' or 'unsatisfied' with access to FSA and this was reflected in the free text comments. Suggestions from GPs regarding FSA included the provision of '…detailed guidelines of when to refer' and patients being given '…advice on non-surgical management…' Comments indicated GP dissatisfaction at the availability and length of wait for surgery. Of those GPs that had patients who had been seen in the Joint Clinic 60% were 'satisfied' or 'very satisfied' with the access to the Joint Clinic. It is not known why 40% were unsatisfied with access to the Joint Clinic.[1] Most GPs were 'satisfied' or 'very satisfied' with overall patient management regarding the Joint Clinic. Free text comments about the Joint Clinic were mixed with negative comments relating to a view that the Joint Clinic was a delay for patients and that the Joint Clinic was seeing people who really required surgery/FSA. However, positive comments noted that patients found the Joint Clinic helpful and appreciated attending.

Conclusion

The majority of GPs were not satisfied with access to FSA but were satisfied with FSA overall patient management. Most GPs with patients that had been seen in the Joint Clinic were satisfied with access to the Clinic, the quality and timeliness of the feedback from the Clinic and the overall patient management by the Joint Clinic.

[1] The waiting time for the Joint Clinic has generally been less than two weeks.

Appendix 3. Southland GP Survey – June 2013

A pre-implementation survey of Southland GPs' satisfaction with the Orthopaedic Outpatient Department service at Southland Hospital. Conducted and reported by Dr Helen Harcombe under supervision of Dr Haxby Abbott..

AIM

To assess GP satisfaction with the service currently provided by Orthopaedic Outpatients for patients with hip and knee osteoarthritis

METHODS

All Southland GPs (n=73) with contact details provided by the Southern District Health Board were invited to participate in the study. GPs were sent a postal survey, a reply-paid envelope and a Management of Osteoarthritis (MOA) pen in May 2013. Approximately 4 weeks later, non-responders were sent another copy of the survey by email. The email also had a link for participants to complete the survey online if they wished. Those GPs without a current email address were sent another copy of the survey by post. The survey consisted of three questions and space for comments. This was the same survey as the one completed by Otago GPs in 2012.

PRELIMINARY FINDINGS

Response Rate

This survey currently has a 63% response rate, with surveys continuing to be returned.

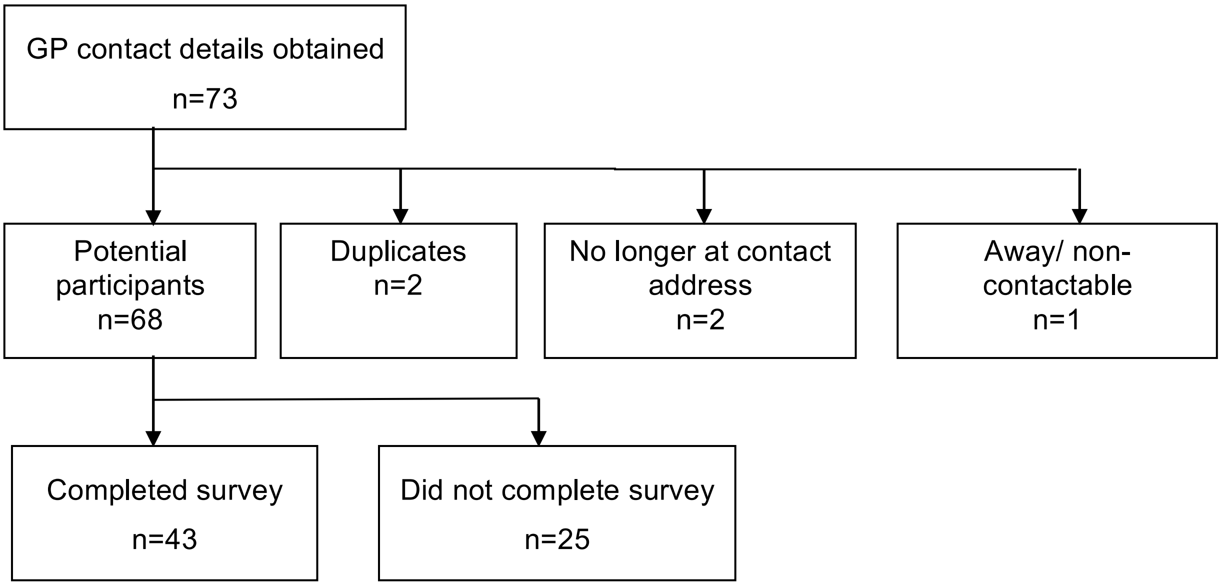

The ease of access (including waiting time) for a First Specialist Appointment (FSA)

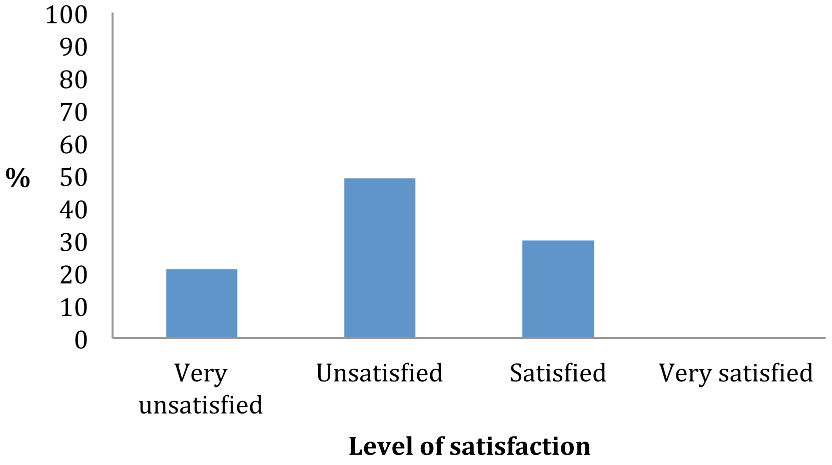

The quality and timeliness of feedback from the appointment

missing data 2% (n=1)

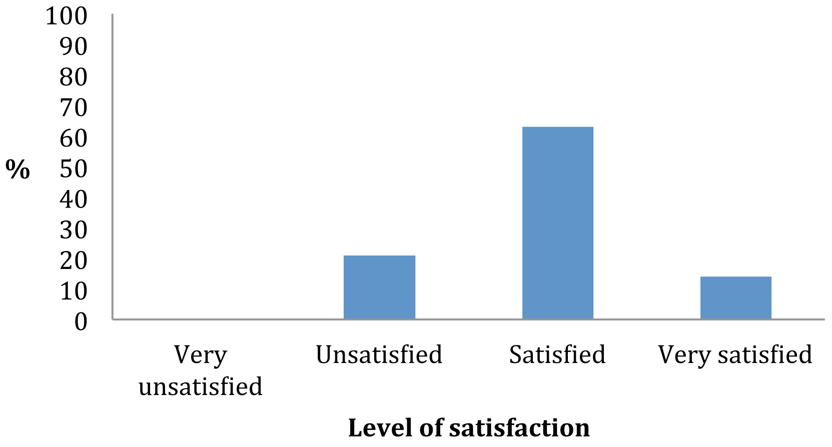

Overall patient management (including treatment plan/implementation, education/advice

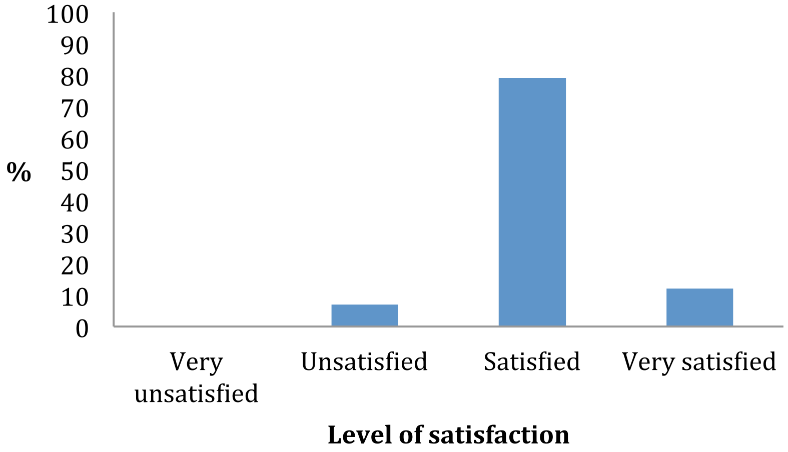

Free text comments (n=20 however some participants commented on more than one aspect)

The ease of access (including waiting time) for a First Specialist Appointment

- "Takes months and months and months. Also they won't even look at the appointment till X-rays are done, these can take months in the public sector too…"
- "Satisfied means I understand but can't be happy about the degree of access"
- "I realise it is beyond your control"
- "I am very satisfied with the treatment providers but very unsatisfied with time taken to obtain treatment i.e. hip/knee replacement"
- "Too slow. 6-12 months to be seen. If seen at all"

Access to other providers

- "Wt loss advice is very difficult esp when specialist suggests the GP does a dietician referral, firstly because there are few things more annoying than being expected to do administration for specialists…, secondly, because the DHB … refuse to accept referrals unless people have an underlying medical condition. Biggest positive we've seen is DHB funding locally based (private) physiotherapy – saves…rural patients travelling for hours or missing out on pre and post op treatment"
- "Would love better access to physio pre-surgery to [illegible] pain and delay the need for surgery"
- [Access to physiotherapy] very poor for OA"
- [Access to weight loss dietary advice] help almost non-existent"

The quality and timeliness of feedback from the appointment

- "Typing pool v slow"
- "Once seen/assessed etc, I appreciate excellent letters (taking on avg 3/52 to arrive) and subsequent care"
- "Don't remember seeing clinic letters very often?? Maybe they are fine, maybe we don't get sent them sometimes"
- "[Unsatisfied] mainly [due to] timeliness"

Overall patient management

- "Depends who they see"
- "Providing motivation to exercise/wt loss would be beneficial. Patients can come back from clinic dejected"
- "Once patient seen – but operation waiting times are ridiculously long – 6-18 months…"
- "The amount of gaming going on through patients talking up their symptoms on screening/scoring phone calls seems sub optimal to say the least. The honest would appear to suffer. Not sure what the solution is though"
- [Quality of care] – generally good once in the system"

Other comments

- "I note and appreciate the clarity of any guideline that seeks to make it very clear as to whom is seen and who is not and surgeons should spend more time operating than chatting and churning out wisdom…"
- "…when they are seen in the private rooms their physical status shows a massive acute deterioration in their physical capacity thus meeting the fairly drastic criteria for TKR/THR soon after"
- "We remain aware that most people come back happy and good after these procedures but not all and this is a normal discussion prior to referral as well as a general assessment of procedure risk etc"
- "The waiting list questionnaire is not always an accurate way of prioritising/scoring patients e.g. use of pain relief, some stoical patients down play their symptoms but can be quite debilitated"
- "Delaying surgery in the elderly can mean they deteriorate further in general cardiovascular/respiratory fitness when their mobility is reduced while on long waiting list causing other issues and costs to the health system"

- "Usually by the time patients have enough points to get on the surgery list they have significant impairment and pain"
- "The 5 month wait is very difficult…"
- "They are getting quite good at getting people in within the MOH guidelines – but this can mean a miserable year for the patient"
- "I cannot discern a difference in prioritisation or referral decline between referrals I label routine or referrals where I express a concern… i.e. it is not apparent to me that referrals are read"
- "When patients interact with the Orthopaedic consultant, they come away with a clear understanding of the management plan. It is not always the same when a patient sees a registrar, and the registrars change with each visit…"
- "OFTEN, the management plan set by the consultant is over-ridden by administrative staff, including letters I have received saying 'Your specialist has recommended surgery but you don't have enough points…"
- "…the patient who is put on the certainty of treatment list, is having their pre-operative assessment soon after seeing the consultant and is being told at this assessment that …doesn't mean that they are even on the list…Patients are confused (as am I) but my other concern is that the health status of the patient can change…"
- "It would be good for an update of the current criteria maybe there is a weblink out there somewhere and that would be fine"
- "I refer to orthopaedic OPD who write and say see physio first; physio then says they need Orthopaedic Outpatients. Why can't physio's put patients back into the system rather than me write again?"

DISCUSSION

This survey had a good response rate and surveys are continuing to be received. The majority of respondents (70%) were 'very unsatisfied' or 'unsatisfied' with the ease of access to FSAs. Free text comments also note dissatisfaction with access to other providers such as dieticians and physiotherapists for patients with hip or knee osteoarthritis. The majority of respondents (77%) were 'satisfied' or 'very satisfied' with the quality and timeliness with the feedback from appointments at Orthopaedic Outpatients. However, timeliness of feedback was raised as a potential area of dissatisfaction in free text comments. Most respondents (91%) were 'satisfied' or 'very satisfied' with the overall patient management. Among other aspects, free text

comments included a desire for guidelines/current criteria and the impact on patients, for example deterioration of fitness, while on waiting lists.

CONCLUSION

Although most respondents were ‘satisfied’ or ‘very satisfied’ with overall patient management, most were ‘very unsatisfied’ or ‘unsatisfied’ with the ease of access (including waiting times) for FSAs.

Appendix 4. Interim Implementation and Process Evaluation – January 2013

The Joint Clinic: Implementation and Process Evaluation of a New Outpatient Service.

Conducted and reported by: Waseem Alzahar, Isaac Campbell, Jacob Edwards, Danial Kim, John Liu, Calum Pears, Natasha Perry, and Shanshan Yan, under supervision of Dr Sarah Lovell and Dr Josie Athens, for Dr Haxby Abbott.

The full document has been submitted to the Orthopaedic Pathway Project management, and also is available from haxby.abbott@otago.ac.nz

For access to the full interim evaluation report, contact Dr Haxby Abbott at: haxby.abbott@otago.ac.nz

Abstract

Aims

The aims of the this study are to assess the implementation of the Joint Clinic proposal made to the Elective Services of the National Health Board, and to assess the process and functionality of the Joint Clinic.

Background

Osteoarthritis (OA) is a common condition in New Zealand. The Dunedin Public Hospital Orthopaedic Department has been overburdened with referrals for assessment of OA patients. The Joint Clinic is a new service operating since June 2012. This is a physiotherapist-led clinic for patients with hip and knee OA who would normally be triaged to be low priority and returned to their GP's care without seeing a surgeon. This study is part of a greater evaluation which will include an outcome evaluation at 12 months.

Method

Data was collected from in-depth interviews with key stakeholders, analysis of the original clinic proposal, observation of the clinic, and review of the clinic's monthly reports. Individuals with a strong understanding of the clinic were sought as participants. Initially, participants were identified from the clinic proposal and a chain sampling approach was taken with initial participants identifying others involved in the clinic to identify more participants. In total, 21 potential participants were invited to take part in the study with 16 participants interviewed in total. All interviews were recorded and transcribed verbatim. Thematic analysis was performed from the transcriptions.

Results

Analysis of the transcripts generated six major themes, including: staffing, appropriate care provision, care coordination, promotion of the service, the Joint Clinic model and Hauora Māori.

With regards to staffing, we found that there was confidence in the competence of clinical personnel and that it was understood that the clinic had the ability to sustain services through recruitment as needed. However, it was found that there was a burden on administrative staff that would need addressing in the form of recruitment and workload redistribution as patient demand increased.

As for appropriate care provision it was found that the Joint Clinic had met its goals of capturing hip and knee osteoarthritis patients who would have otherwise been returned to the GP without assessment. This was reflected in the markedly reduced numbers recorded in the Joint Clinic's monthly reports. Furthermore, it was found that the clinic was running under capacity with the potential to serve more patients than what was currently coming through the programme. Leading on from this there were concerns regarding the longer than planned appointment times of the clinic. Sessions of 45 to 60 minutes were possible under current patient loads, however, it was unsure if this was practical into the future, especially if patient volumes were to increase. The overall impression of the Joint Clinic from interviewees was positive.

In terms of the continuity of care and its coordination it was found that the referral process had been implemented as outlined in the initial proposal. There are avenues being explored to alter this process to increase the number of patient influx. The model of care offered by the Joint Clinic was generally well-accepted and acknowledged by key stakeholders. The timeliness of appointments after referral was between 2 and 3 weeks, which is consistent with original plans. Communication was reported to be functional and efficient between both internal and external stakeholders with information and feedback shared between all concerned.

There were issues in promoting and increasing the awareness of the Joint Clinic due to a potential overload of incoming referrals. Initially, GP's were informed of the clinic's existence through a GP CME meeting but there has not been any active promotion of the service since. Despite this it was found that awareness of the Joint Clinic was good with most stakeholders interviewed having a solid understanding of how the clinic worked. However, there was some confusion among certain interviewees with regards to the referral process and the exact service

that the clinic offers.Due to the aforementioned issue of the clinic running under capacity it was the suggestion of some interviewees that the scope and model of the clinic be altered for expansion and to allow more referral pathways.

No specific methods to target the Māori population were incorporated in the original proposal but after the interviews it was found that more information sharing and involvement with Māori liaisons would allow for better accommodation of Māori patients coming through the service.

Conclusion

Overall the Joint Clinic has been implemented as proposed. It is functioning well to capture the intended population and is operating as planned to relieve the load on the orthopaedic department and grant more patients with assessments of their conditions. In general, stakeholders have a positive view of this service as it is running currently due to most if not all aspects of the clinic being implemented as planned. However, as the clinic ages and looks to expand, new considerations must be taken in to account to modify the current model. For example, there is potential for expansion through GP referral, different pathologies, surgical post-operative follow up, and physiotherapist triage (as opposed to by orthopaedic surgeons in the current model). There are plans to carry out an outcome evaluation of the service at 12 months, which will help in identifying potential areas for improvement and yield suggestions for clinic model alteration. Any significant changes to be made to the clinic should await the results of this outcome evaluation as this study only focussed on evaluating the implementation and the process of the clinic.

Appendix 5. Interviews with Staff and General Practitioners – April-May 2013

A qualitative evaluation conducted and reported by Dr Helen Harcombe under supervision of Dr Haxby Abbott.

AIM

The aim of this process and outcome evaluation was to explore the functioning, perceived effectiveness and overall operation of the Joint Clinic.

METHODS

Six Southern District Health Board (SDHB) staff and seven General Practitioners (GPs) were invited to take part in a one-on-one in-depth interview. SDHB staff invited to take part were those identified as being closely involved with the Joint Clinic. GPs were semi-randomly selected from those GPs referring patients that had had a follow-up appointment at the Joint Clinic. Selection was semi-random to ensure that each GP interviewed was from a different practice. Consent was recorded verbally at the start of the interviews and on a written consent form. Approval was gained from Health Research South to undertake interviews with SDHB staff and the study had ethical approval from the Lower South Regional Ethics Committee.

Interviews were digitally recorded and transcribed by an independent transcription company. Interviews were semi-structured with a topic guide, based on domains of inquiry suggested by Hollander et al [34], that was slightly adapted for each group of interviewees (GPs, Joint Clinic staff, Other SDHB staff). Transcriptions were checked against the interview recordings by the interviewer and corrected if necessary. Thematic analysis was carried out based on the Framework Method Ritchie and Spencer, 1994 cited in [35]. This involved stages of familiarisation, identification of a thematic framework, indexing, charting and mapping and interpretation. NVivo software version 10 [36] was used to organise the data.

In places in this report, quotes from participants have been included. Participants have been given a unique identifying number and, at times, square brackets have been used and words inserted either to help readability or to alter wording to more generic, less identifiable terminology.

RESULTS

With the exception of one GP, all those invited to be interviewed agreed to participate. Interviews were between 8 and 31 minutes long and took place either at Dunedin Hospital or at GP practices between April 8th and May 7th 2013. The final thematic framework had 26 content codes which were organised into 6 themes: impact, the value of the Joint Clinic, access, knowledge and understanding of the Joint Clinic, communication, and the future. Several codes did not fit within the 6 themes and are discussed separately.

Theme 1: Impact

Main effects of Joint Clinic

The main effects of the Joint Clinic were generally seen to be positive. Positive effects identified were that patients who would previously have been returned to their

GP were now being seen at a secondary level. It was hoped that this would reduce the burden on orthopaedic outpatient service. After being seen at the Joint Clinic patients were thought by interviewees to be in a better physical state with increased confidence, increased quality of life, increased strength and general fitness and with improved self-management. In contrast, the idea was raised that the main effects on patients were psychological, rather than having a functional effect. It was seen as positive that patients perceived that they were being attended to and that they were being seen and listened to rather than being ignored. Professional development and the creation of new roles for Joint Clinic staff were also noted as positive effects of the Joint Clinic initiative.

When specifically asked if there had been any negative impacts of the Joint Clinic the idea was raised that there were no negative effects; "I don't think there can be any negative effects of attempting to help someone with a chronic pathology, no" (P1, SDHB). However, a negative effect of the Joint Clinic that was raised was an increased workload for several staff members, one of which, (administration) had resolved. The other was for the orthopaedic consultant involved with the Joint Clinic staff and, although this workload was expected to lessen over time, the turnover of physiotherapists (three within one year) had increased the workload thus far. It was raised that the type of patients that were seen at Joint Clinic seemed to be more severe than had been anticipated at the outset. The idea was also raised that the Joint Clinic had meant an increase in time delays for patients to see an orthopaedic consultant. Intra-departmental politics were briefly noted (the nature of this was not expanded upon any further). Several potential or hypothetical negative impacts were mentioned; there was the potential that "…patients might feel fobbed off if the purpose of the Joint Clinic has not been explained to them" (P3, GP) and it was also noted that, hypothetically, it would not be good if a potential effect of having the Joint Clinic was that surgery numbers/funding was decreased.

<u>How the existence of the Joint Clinic has changed the service previously offered</u>

In general, participants felt that the Joint Clinic had enabled people who would otherwise have been sent back to their GP unseen, to be seen by a health professional at secondary level and offered non-surgical interventions; "…in the past of course, GP referrals have been triaged by the orthopaedic consultants. If they deem not to be able to help them, they then have sort of been put into a big black hole back to the GP in many ways, and a lot of them have just been left floundering" (P12, SDHB) and; "Well the idea was to get more patients seen…because we have an issue with access to the outpatient first specialist appointment" (P5, SDHB). However, the idea was raised that perhaps the Joint Clinic was not just seeing those patients that would otherwise have been sent back to their GP. In line with this, one GP felt the Joint Clinic had slowed things down for patients as they were being referred for a First Specialist Appointment (FSA) only after being seen for a follow-up Joint Clinic appointment, rather than going straight to FSA. It was hoped that "…the Joint Clinic will look after the patient… more quickly, more efficiently, and more to the point…and help GP[s] to, to manage a long term problem" (P9, GP). Psychological benefits were noted in that "…patients do have the perception that they, that something's happening" (P10, GP). However, functional effects in changing the service previously offered were also noted; "…it's absolutely plugged a huge gap.... And that ultimately it would be nice to think that it might defer surgeries for some people or at least when they do come to surgery, they'll actually, their post-op recovery will be a lot better" (P12, SDHB).

Impact of the Joint Clinic initiative on patients

The perceived impact of the Joint Clinic on patients ranged from being extremely positive to mixed, unsure or negative. However, the general feeling was of a positive impact. Positive impacts were felt to be that patients had explanations of their pathology, assessment and advice. Patients were able to have interventions that improved their self-management, strength, function, weight management and general fitness. Patients were listened to and did not feel forgotten or left by the system and had "…the perception that something's happening" (P10, GP). For example; "All of them [patients] have had an improvement in their function. That doesn't translate into leading, needing less pain relief. It doesn't translate into not needing joint replacement. It does translate into believing that they haven't been abandoned by the system, into realising that they will recover from what is major surgery and holds considerable fear for most people still" (P3, GP). Patients were felt to have an increased understanding of their condition and increased feelings of control. It was, however, noted that initially patients may feel frustrated that they had not been seen by an orthopaedic surgeon. Others may "…come away feeling a bit despondent" (P8, GP) if "…suddenly at the Joint Clinic they're told they have to lose weight" (P8, GP). The idea was raised that the Joint Clinic may provide a "band aid" (P7, GP) and that what the patients really required was surgery. The idea was raised that attending the Joint Clinic may have expedited the time to FSA for some patients; "… [the Joint Clinic] gave us a pathway to get into the orthopaedic system. I would like to think a little bit faster than if they had just been out at GP level again" (P12, SDHB). In contrast, a GP felt that the Joint Clinic had actually delayed the time to FSA. Others felt the Joint Clinic probably had had a mixed impact for patients; "Some people will have benefited from it, other patients might have just had their orthopaedic management delayed and hopefully some of those that do need surgery will have been picked up and triaged into an earlier FSA" (P4, SDHB).

Impact on orthopaedic consultants/clinics

There has been an increased workload for the one orthopaedic consultant directly involved with the Joint Clinic. Participants noted that an effect on orthopaedic clinics as such would not necessarily be expected as the patients seen in the Joint Clinic were ideally those that would otherwise have been returned to their GP and would not have been seen by an orthopaedic consultant anyway. However, the idea was raised that if orthopaedic consultants were only seeing those patients who reach the clinical threshold, but not necessarily the financial threshold for surgery, the actual proportion of their patients who may be unhappy because they cannot be offered surgery may increase.

Referrals sent back to GPs

Prior to the existence of the Joint Clinic, some patients were being sent back to their GP without being seen; "…well, instead of the referrals being triaged and sent back to the GP, not being seen at all… they're now being seen" (P1, SDHB) and; "Well I think it's [the Joint Clinic] a good idea. It's much better than you get the blanket letter, 'we can't expect to see you within six months so therefore we won't see you.' Which was, seemed to be happening before that clinic existed" (P10, GP).The idea was raised that there had been a reduction in return referral to GPs since the Joint Clinic had been in place. In contrast, the idea was also raised that perhaps there had been an increase in return referral to GPs. However, on reflection it was thought that this may have been for other regions/pathologies such as shoulder conditions and not necessarily for patients with osteoarthritis (OA) of the hip and knee. However, the

idea was also raised by some GPs that they had not noticed a difference in referrals returned to them from orthopaedics since the Joint Clinic had been in place because they were confident that the patients they did refer were appropriate and would be seen. It was also noted that GPs were referring people that they felt should be seen by an orthopaedic consultant; "…the thing from my end, from the GP end, is that by and large we are referring people where we truly think there is a justification for them to be, being assessed for replacement" (P10, GP).

Meeting unmet need

Of those who were able to comment on whether they thought the Joint Clinic was meeting or addressing unmet need there was a general agreement that it was. However, this had a number of qualifications. One participant was careful to say that; "it's helping the demand for FSA which it was, is also in excess of what we could supply" (P4, SDHB) while another felt that although the Joint Clinic was meeting the need of the patients currently referred to orthopaedic outpatients, the Joint Clinic was not necessarily meeting the needs of those with early OA as these patients were not currently referred to orthopaedic outpatients. Another participant noted that there was the potential for the Joint Clinic to "…reach even greater numbers of the population" (P12, SDHB).

Theme 2: The value of the Joint Clinic

GPs generally felt the Joint Clinic was a positive initiative and was of value; "I think it's enormously valuable" (P3, GP), "Well I think it's a good idea" (P10, GP) and; "I think it's certainly got a place (P12, GP). However, the idea was raised that, if the clinic was seeing people for whom everything had already been tried, then it was not of value. It was felt the Joint Clinic would be of value if it was seeing patients with early osteoarthritis. Others had the impression that GPs were generally supportive of the Joint Clinic; "…the GP's are definitely coming on board too. Because, I mean on their referrals they're actually, quite a few of them are very proactive in writing that they think their patient would be suitable for the Joint Clinic" (P2, SDHB).The staff of the Joint Clinic were seen to do a good job and provide a good level of care; "I think the allied health team do a really great job with it" (P2, SDHB).

Participants had the impression that patients were generally appreciative of the Joint Clinic and felt that it was of value; "Most of my patients would be enormously grateful for the care they receive. All of them have had an improvement in function" (P3, GP), "…my patients, they are happy" (P9, GP) and; "patients who have been there give positive feedback" (P7, GP). However, the idea was raised that not all patients saw value in the clinic; "They like meeting the people, but it hasn't helped their hip" (P8, GP) and; "From their perception it's not ideal but they've been listened to" (P10, GP). Patients were seen to appreciate being seen promptly; "they're quite excited the fact that they don't wait long to see somebody" (P2, SDHB). However, the idea was also raised that some patients could be disappointed not to see an orthopaedic consultant; "There are some patients that are initially quite upset or potentially frustrated with actually the fact that they're not seeing an orthopaedic doctor. However, I think with just a little bit of explanation of what that clinic actually involves, I think they realise that what the clinic has to offer is really, is really quite beneficial for them" (P6, SDHB).

Theme 3: Access

The short waiting time to be seen in the Joint Clinic was viewed positively; "with regards to waiting times and accessibility, they're [the patients are] all very happy with it, because ...they're not really waiting at all for an appointment" (P1, SDHB) and; "It appears to me that they get early appointments from Joint Clinic rather than from you know what do we call, classical departments" (P9, GP). The idea was raised that GPs could potentially refer more patients, and more directly, to the clinic; "…obviously what would be really good would be to get more GPs on board and get them to refer straight to ... the clinic, realising that, you know, [it is] focusing on conservative management" (P6, SDHB). This was echoed by a GP; "My one consideration is that I would like very much to be able to make that referral directly" (P3, GP). It was noted that the Joint Clinic could be accessible to more people; "…in fact Joint Clinic should be much more widely available than it is. I think there are many patients who would benefit from access to Joint Clinic possibly up to a year before they would actually be requiring surgery" (P3, GP).

Participants had mixed views on the current referral system to the Joint Clinic, (patients are referred to orthopaedic outpatients and then triaged to be seen at the Joint Clinic). As noted previously, some GPs would like to be able to refer directly to Joint Clinic and in fact some had already written that they would like their patient to be seen at the Joint Clinic on their referral; "…in some instances I've actually referred, directly addressed it to the Joint Clinic as opposed to orthopaedic surgeon" (P10, GP). Some would like to have a separate referral system for the Joint Clinic. However, others were happy to have it as is; "…by and large we're sending the people where we think that there's probably is going to be, need to be a joint replacement… so I don't think it matters too much if I'm referring directly to orthopaedics or directly to Joint Clinic" (P10, GP). The current referrals to orthopaedic outpatients were thought to be having an impact on the type of patients being seen at the Joint Clinic; "…just due to the type of referrals that [the Clinic is] getting from GPs now, they're holding onto their patients a lot longer, and so therefore, by the time [they are seen] at clinic I think they're a lot worse than what was probably initially hoped for" (P6, SDHB). It was felt that GPs could send patients with less severe symptoms to the Joint Clinic although it was acknowledged that this would be likely to impact on the number of referrals to orthopaedic outpatients; "I think that's more of an educational thing with the GPs, rather than waiting for the patients to be quite severe and then referring them to see a surgeon, I think that it would probably be quite a good idea to educate them with regards to, if someone has some, you know, moderate osteoarthritis, to send them in as well. That will certainly increase the numbers through the outpatients, which may not be a good thing, but I think, in terms of the patient population, I think it would be very good for them" (P1, SDHB).

The 'virtual triage' process was seen as an important aspect of the Joint Clinic; "I think what's very important is the selection of patients which are seen in the Joint Clinic..." (P5, SDHB). It was acknowledged that this could be difficult if there was insufficient information on the referral. Most patients referred were thought to be appropriate. However, there were other potential problems identified with the process; "...not all the referrals are actually read correctly and X-Rays are looked at" (P6, SDHB) and; "I'm not really sure if all the consultants are actually necessarily referring the patients on to the Joint Clinic" (P6, SDHB). Another potential issue raised was that there may sometimes be a delay in the triaging, or returning of the referrals to be booked, which it was thought may result in gaps in the flow of the clinic bookings. It was hoped that the system meant that if patients who actually would be appropriately seen by an orthopaedic surgeon were triaged to be seen in the Joint

Clinic that the Joint Clinic staff would then refer the patient to the orthopaedic consultants; “I would hope that if they get directly to Joint Clinic and, and they’re graded at surgical, that they’ll get that next step (P10, GP).The idea was raised that, from a GP perspective, there was a lack of understanding of the criteria for the triaging to the Joint Clinic; “I have no idea what criteria they use to decide whether my referral to orthopaedic outpatients results in a subsequent onward referral to the Joint Clinic or to orthopaedic outpatients. I have the impression, that may be wrong, but just that they all get pushed that way” (P8, GP).

Theme 4: Knowledge and understanding of the Joint Clinic

The idea was raised that there had been an increased understanding of the Joint Clinic by consultants, GPs and other DHB staff as time passed; “...they’re [the consultants are] understanding more what the joint clinic’s about and how it’s benefitting the patients...” (P2, SDHB) and; “...now that people understand what the Joint Clinic is actually trying to achieve, and, both the GPs and [DHB staff] as well...” (P2, SDHB).However, it was noted that there may still be a lack of clarity and understanding about the Joint Clinic; “I think the perceptions of what the Joint Clinic’s trying to achieve or is actually doing differ across the primary care, secondary care sort of interface. So I’m not sure it’s, people are totally clear about what’s happening” (P4, SDHB).

The idea was raised that it may have been helpful for staff to know a bit more about what was happening with the Clinic earlier on, for example what the patients thought of attending the Joint Clinic. It was noted that early on there may have been a lack of understanding by DHB staff of the purpose of the Joint Clinic although this was felt to be improving. There was a general sense that because the data had not yet been analysed it was difficult to ascertain what the outcomes were. A lack of clarity/understanding was noted about the exact role of the nurse and the physiotherapist. GPs spoke about being unclear about exactly which patients were being sent to the Joint Clinic, what the virtual triage criteria was, what happened when patients were seen at the Joint Clinic, which practitioners were involved and what was actually being offered in comparison to their understanding of the Clinic. It was also raised that the existence of the Joint Clinic may not be well known among GPs. It was noted that it was very important that patients coming to the Joint Clinic had a clear understanding of the type of practitioners they would see.

Theme 5: Communication

Communication was noted as being very important in an initiative such as the Joint Clinic; “...communication is very important on all levels. As to whether, whoever you’re dealing with as far as staff and colleagues as well as then obviously communicating with your perceived source of referral. And ensuring everybody understands why you’re there and what you’re trying to achieve” (P12, SDHB). The idea was raised that the communication and liaison between the many people involved in the Joint Clinic was satisfactory; “I think it’s working reasonably well” (P4, SDHB) and; “I think it’s been quite good” (P2, SDHB). This was also felt to be reasonable in specific areas such as liaison about specific patients, between Joint Clinic staff and orthopaedic staff and between the Joint Clinic staff themselves; “I think communication on individual patients at a personal level is pretty good” (P4, SDHB) and; “there’s a lot of trust and respect there within that relationship [between staff members]” (P6, SDHB). However, there were other areas where it was felt that communication could be improved; “I don’t think there’s been good communication

about progress, about numbers and what it's achieving" (P4, SDHB). It was acknowledged that access to orthopaedic staff was on an informal basis for Joint Clinic staff however this was working well. However, another participant felt this could be improved; "I think that link should be developed and be a lot clearer..." (P11, GP).

In the early stages, it was felt that communication between the University and the DHB could have been improved and it was suggested that this may have been affected by the Joint Clinic starting earlier than some other projects involved in the Orthopaedic Patient Pathway project (OPP). This communication was felt to be improving. It was also acknowledged that there were a number of meetings where it was possible for any issues to be discussed.

Letters to GPs about patients from the Joint Clinic staff were generally felt to be of a good standard and thorough; "...you know well it's a substantial letter" (P10, GP). However, a contrasting view was also raised; "I think the letters back are pretty superficial. It wouldn't, the letters for example do not teach me anything that I might use with my patients before they got to Joint Clinic" (P3, GP). The idea was raised that progress reports would be useful for GPs and also that electronic communication would reduce any delays in GPs getting letters from Joint Clinic staff.

Theme 6: The future

Advice to others setting up a similar service was to start small and then build up; "I think they do have to do what we did and start slowly…" (P2, SDHB). It was felt it was important to know what the benefits of the Joint Clinic were and that the Joint Clinic would be appropriate to meet the needs of a different place. A team approach was valued; "…GPs need to be involved as well and it's the whole, the whole, you need to look at the whole patient journey, not just that, not just at the clinic in isolation. You need to look at the links, how it links in with the, the GP, how it links in with the orthopaedic surgeons and things like that. It can't be set up in isolation, you have to have a, basically the whole team has to be involved" (P5, SDHB). Continuity of the physiotherapist in the Joint Clinic was raised as important. Communication was recommended so that there was a clear understanding of the patients that would be seen and the purpose of the clinic. Before a new clinic was set up elsewhere it was felt that it was important to evaluate the Joint Clinic. It was also noted to be important to evaluate the unmet need in a different area, for example by a pre-implementation survey of GPs as was done in Otago prior to the setting up of the clinic. Staff training was mentioned as being important. However, with the recent changeover of physiotherapists it was felt that maybe more time needed to be taken before staff would be ready to train others; "I don't think we're ready yet to be able to move, to move the clinic on to other sites yet until the physiotherapist is feeling quite, yeah, is feeling like they're ready to educate and give advice to other DHBs" (P6, SDHB). Modelling it on the current clinic was advised; "I guess you'd want to model it fairly closely on what we know… model it fairly closely on what we know works well here" (P1, SDHB).

When considering the possibility of similar clinics being set up elsewhere, participants reflected on what they might do differently if they were setting up the Joint Clinic again. The idea was raised that the Joint Clinic had been well set up. It was seen as being evidence based practice which was important. It was noted that it was good that it was a joint project between the University and DHB; "I know a lot of hard work was put in, a lot of people and because, as I said before, you had more acceptance within sort of, particularly with the University and the Hospital as a joint

project" (P12, SDHB). In contrast it was raised that if setting the Joint Clinic up again it could be done without the University involvement and could be set up very quickly. However, others noted that starting small was important; "...I think it all needed to be sort of tested, the waters needed to be tested, and it all needed to be felt out. And I think that certainly was the best way to do it, start with smaller numbers and then build yourself up, and really get a feel for what needs to be done in the clinics" (P1, SDHB). If it was being set up again the idea was raised that it would have been good if all the projects in the OPP had started at the same time.

The Joint Clinic was felt to be working well; "…in terms of the content of the clinic, and how it works with the nurse and the physio, I think, is running very well" (P1, SDHB). However, participants were specifically asked how they thought the Joint Clinic could be improved and this raised several suggestions. Continuity of staff was an area that could be improved as there had been a turnover of physiotherapists. Increased cover for the physiotherapist was also suggested. Having clear discharge criteria was also noted as an area that could be improved. It was also suggested that the nurse could be involved in the follow up clinics as well as at the patient's initial appointment. Increasing the awareness of the clinic among GPs was an aspect that it was suggested could be improved. In addition, providing additional information about exactly what is offered at the Joint Clinic would be helpful for GPs. The idea was raised that progress reports to GPs would also be helpful. Raising the profile and value of the Joint Clinic among DHB orthopaedic staff was also mentioned; "…that it's prominence is probably put out there a little bit more and that it's looked upon as being a valued part of particularly the orthopaedic department" (P12, SDHB). Increased clarity of the patients that the Joint Clinic is focussing on could be improved; "I think there needs to be clarity at least for me, I don't know about my colleagues, in terms of who they really should be focusing on" (P8, GP). It was felt the Joint Clinic had the most value for those with moderate osteoarthritis. The idea was raised that the Joint Clinic was still establishing itself; "So the Joint Clinic needs to establish its place. So I think it definitely has a place, I still think it's trying to find what that is" (P8, GP). Increasing the numbers of patients with moderate osteoarthritis seen in the Joint Clinic was suggested. However, it was not clear whether this was primarily being affected by the types of referrals to orthopaedic outpatients by GPs or whether it was being affected by the virtual triage system; "…by getting more of the people, who [the Joint Clinic] can make a difference with as well. And that may not necessarily be the triaging. It may be the GPs referring in…" (P1, SDHB).

To ensure the future viability of the clinic it was suggested that it was important for the funding to be maintained. It was also suggested that the consultants needed to have increased awareness of the clinic and that it's build up was gradual; "…making sure that it's gradually built up, it's, we don't have, you know, everything forced on it at once, 'cause then it'll fall down. I think it's important that it's just slowly propped up, scaffolded, so that there's, you know, there's a strong base, basically" (P1, SDHB). Communication and working together was thought of as important; "I think definitely it's the communication with everybody involved. Everybody needs to be on the same page, and I think everybody needs to understand that each of the people that have got a vested interest in it come from different professions, they all have their own ideas about what the joint clinic needs to be doing. And I think it's more important that we work, I guess how would you, in the perfect world, we're all working together for the same interest…" (P1, SDHB).

In the future it was felt that the Joint Clinic could be expanded. Several ways it could be expanded were suggested. Suggestions were increased hours so more patients could be seen and the development of satellite clinics in rural areas. It was suggested that it could also play a role in providing advice and monitoring of patients who were on active review and it could expand to see different joints and/or pathologies; "If one accepts that around about 25% of all general practice consultations relate to the musculoskeletal system, it would seem pretty self-evident that we are currently restricting intervention to people needing hip and knee replacement…" (P3, GP). It was also suggested that it could be altered to be a combined clinic with orthopaedic consultants however it was acknowledged that this would be likely to have a negative impact on the waiting list. It was noted that it was important that the Joint Clinic did not duplicate what could be carried out by GPs and that it was important to clarify the role of the Clinic.

Other aspects

Several other aspects were raised that did not necessarily fit within the six themes. It was felt important that there was not a duplication of the roles between the physiotherapist and the nurse. However, it seemed that these roles were now working well; "I think now they're [the roles are] quite defined and quite separate. Initially, I think there was quite a lot of overlapping. However, as time's gone by and obviously when you work with [the healthcare professional] for a lot longer there's a lot of trust and respect there within that relationship, so I don't think there is any, a lot of crossing over now at all" (P6, SDHB). It was noted that there was no cover for the nurse involved in the Joint Clinic and that cover for the physiotherapist could also be increased.

Staff working in the Joint Clinic noted that being involved in the Clinic had been a good experience; "I've really enjoyed working in the clinic. I think it's a great opportunity" (P6, SDHB). Staff appeared to have enjoyed working together; "…certainly working with the [other provider] in close association with that is very good" (P12, SDHB). The opportunity provided by the Joint Clinic to extend their skills as providers was welcomed by Joint Clinic staff. There was mixed acceptance of the term 'physio-led' Joint Clinic with the idea raised that this was both appropriate and not appropriate.

The length of time for the appointments in the Joint Clinic was now 45 minutes and this was thought to be appropriate although it was suggested that 15 additional minutes for paperwork could be used prior to seeing the next patient. The idea was raised that the role for Joint Clinic staff worked very well as a part-time role. However, due to the nature of the position, it was suggested that the role would be less desirable if full-time.

The training program that was provided for Joint Clinic staff was seen as being helpful. Additional training that participants suggested would be useful were regular sessions to provide professional development with regard to reading X-Rays and the addition of formalised ongoing training sessions. Continuing to attend some orthopaedic clinics and possibly seminars such as those run by the Osteoarthritis Research Society International (OARSI) were also raised as suggestions for ongoing professional development. It was noted that it was important for the 'back-up' physiotherapist to continue to have ongoing involvement with the clinics to maintain

awareness of the running of the Clinic and the necessary skills. This involvement also provided a form of peer review for the physiotherapists working in the clinic. The idea was raised that this was currently happening on an informal basis but that this could be more formalised.

DISCUSSION

In general, the Joint Clinic appears to be perceived as having a positive impact on both patients and staff. It is a valued initiative and is seen to have improved the service previously offered to patients. It was felt that patients who would not otherwise have been seen at a secondary level were able to be seen and that there was very little waiting time to be seen in the Clinic. There were some negative effects discussed and ways that the Joint Clinic could be improved were suggested. It appears that one of the main areas for improvement is the need for a clear understanding of the purpose, process and type of patients to be seen at the Joint Clinic. Having now been operating for 12 months, there appears a need for renewed clarity around the purpose and scope of the Joint Clinic and this needs to be effectively communicated to all those involved.

One of the key concerns appears to be a tension between the scope/type of GP referrals to orthopaedic outpatients and the type of patients that potentially may benefit most from the Joint Clinic. It is possible that the tension between these potentially differing populations may increase pressure on orthopaedic outpatients if it is not carefully managed but may also limit the effect that the Joint Clinic could have. For example, if the Joint Clinic was more widely ‘advertised’ as being able to see patients with moderate OA hip and knee, this may generate a greater number of referrals to orthopaedic outpatients of patients who are not at the stage of needing to be referred to an orthopaedic surgeon. Those with moderate OA would potentially be returned to their GP without seeing an orthopaedic surgeon. With the initiation of the Joint Clinic they may be triaged to be seen at the Clinic. However, there could be unrealistic expectations from patients that they should/would see an orthopaedic surgeon and an increased flow of patients through orthopaedic outpatients. A related aspect is that GPs are currently referring patients to orthopaedic outpatients who they feel should be seen by an orthopaedic surgeon. The tension between these aspects will need to be resolved when considering the future of the Clinic. Being very clear about the purpose and scope of the Joint Clinic and communicating this to all involved may help with this aspect. The related aspect of triaging the ‘correct’ patients into the Joint Clinic seems of critical importance likely to impact on both patient outcomes and also on how the Joint Clinic is perceived and valued. Appropriate triaging ties in with being clear about the purpose and scope of the Joint Clinic and it may be helpful to clarify this both to those undertaking the triaging and to those who are referring into the system.

Once an evaluation of the effect of the Joint Clinic on patients has been completed, the findings should be communicated to GPs so they are informed about the potential benefits for their patients. An increased awareness and understanding of the Joint Clinic by GPs seems important at this stage. However, this also needs to align with a closer consideration of the scope of the Clinic.

In relation to operational aspects of the Clinic, ensuring that there is adequate cover for both the physiotherapist and the nurse seems important. It also appears that the

workload for the orthopaedic consultant involved in the Joint Clinic may need to be examined to ensure that it is manageable and if not, that ways of addressing this are looked at. Formalising and extending the ongoing training for Joint Clinic staff could also be considered.

Suggested future directions of the Joint Clinic include an expansion of the current clinic or beginning similar clinics in different settings. A consideration of the merit of these is beyond the scope of this project and will need to be examined separately.

One of the strengths of this qualitative evaluation is that it was carried out by a University staff member who has not otherwise been involved in the set-up of the Clinic. The interviews specifically sought out both negative and positive aspects. Interviews were semi-structured with questions asking about specific areas of interest to the researchers. However, there was also scope for the participants to discuss aspects that were important to them that were not covered within the topic guide. A potential limitation of the research is that participants may not have felt able to discuss all aspects freely. Participants were advised at the start of the interviews that, although data would not be identifiable by name, it was possible that the content may mean they could be identifiable to colleagues due to the nature of the Clinic and the small number of people directly involved with it.

CONCLUSIONS

There are many aspects of the Joint Clinic that seem to be working well. Having been in operation for 12 months it seems appropriate, in line with this evaluation, to re-evaluate and clarify the scope of the Clinic and to communicate this effectively to those involved at each stage of the process. The findings of this qualitative research should be taken as one part of the 12 month evaluation but provides aspects for consideration as the Joint Clinic moves into the second year of operation.

Acknowledgements

Thank you to all those who participated in these in-depth interviews.

Appendix 6. Joint Clinic Patient Survey – April 2013

A postal survey conducted and reported by Dr Helen Harcombe under supervision of Dr Haxby Abbott.

AIM

To investigate patient satisfaction with the Joint Clinic.

METHODS

All patients (n=66) who had been seen for at least one follow-up appointment at the Joint Clinic by early April 2013 were invited to participate in a postal survey. Potential participants were sent a letter of invitation, the postal survey and a reply-paid envelope. A copy of the original information sheet and consent form that they had completed at the time they were first seen at the Joint Clinic was included with the letter as well as a Management of Osteoarthritis ('MOA') branded pen. Two weeks later non-responders were sent a reminder letter and another copy of the survey.

The postal survey consisted of 11 questions assessing: satisfaction with the overall experience of attending the Joint Clinic, the waiting time to be seen at the Clinic, knowledge and expertise of Joint Clinic staff, treatment provided at the Joint Clinic, physiotherapy outpatients and other treatment such as that provided through orthotics or dieticians, how satisfied patients were to be seen by Joint Clinic staff rather than an Orthopaedic Surgeon, how satisfied they would have been to see Joint Clinic staff if they knew that they would not see an Orthopaedic Surgeon at all, the main effect of attending the Joint Clinic, the overall outcome from attending the Clinic and whether or not they would recommend the Joint Clinic to others. Several of the questions also allowed for free text comments.

Descriptive analyses were undertaken. Where few free text comments were provided, these have all been included in the results. Where a larger number of free text comments were provided, the main ideas have been summarised and a selection of quotes that illustrate the ideas raised have been included in the results. At times, within the quotes, square brackets have been used to enhance readability or to reduce the likelihood of participants being identified.

This project was approved by the Lower South Regional Ethics Committee.

RESULTS

Of the 66 potential participants, one invitation was 'returned to sender' as the patient was not at the contact address. Of the 65 potential participants remaining: 5 declined, 44 completed the survey and 16 did not respond. Of those who declined, 3 were unable to complete the survey due to age or health issues. This gave a response rate of 71%. Respondents had a mean age of 69 years and 48% were male.

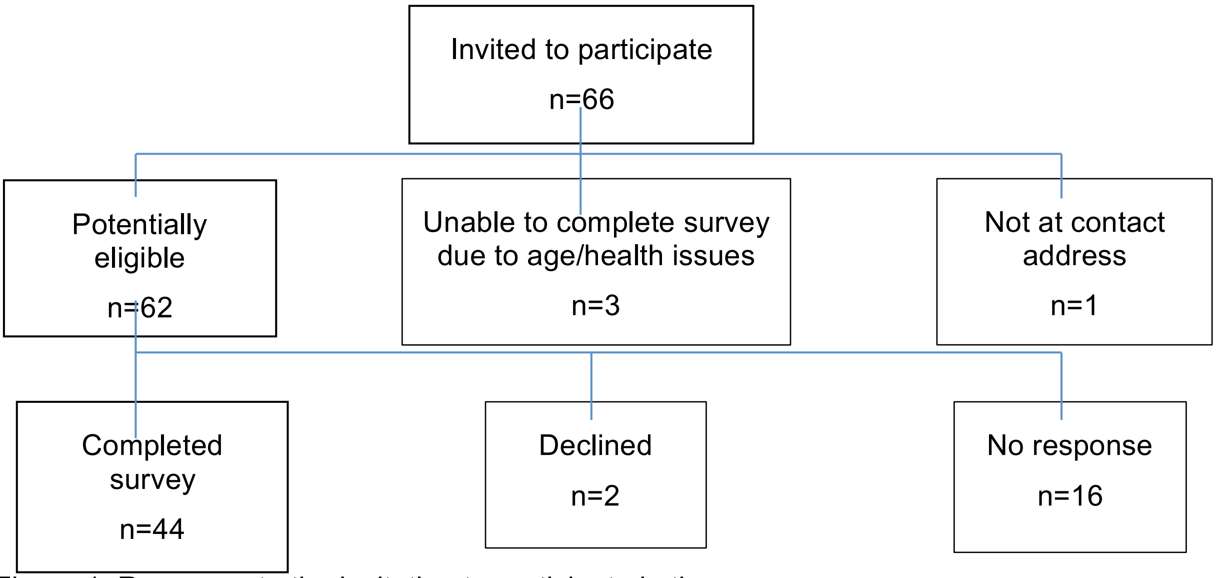

Figure 1: Response to the invitation to participate in the survey

1. Overall experience

Overall, how did you find the experience of attending the Joint Clinic? (Consider your interaction with staff, view of the service offered, timeliness of the service offered)

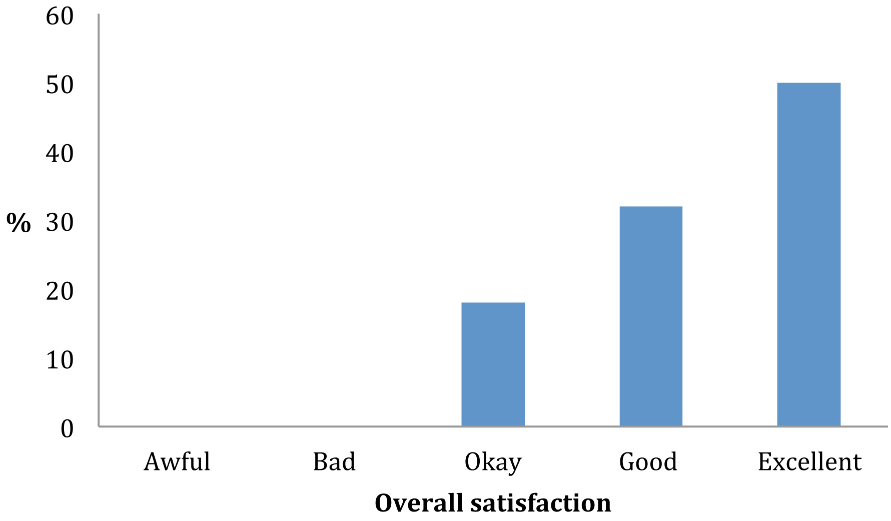

Figure 2: Satisfaction with the overall experience of attending the Joint Clinic

1a. Comments about the overall experience of attending the Joint Clinic (n=5)

Participants commented positively about the staff of the Joint Clinic and the service provided. However, difficulty in getting to the Joint Clinic was raised and an expectation that conservative treatment would not help was also noted:

- "Staff all very pleasant and helpful. Service offered excellent. Good advice given…"
- "Clinic and staff – considerate and helpful."

- “[The physiotherapist] was helpful and informative. Receiving walking stick for support was very appreciated as it also helped my good knee and hips.”
- “The staff are very nice and friendly. But when you have to rely on other people to get you there it isn't very convenient.”
- “I told the physio before he started that physio won’t fix worn bones.”

2. Waiting time

*How satisfied were you with **the length of time** you had to wait for an appointment at the Joint Clinic?*

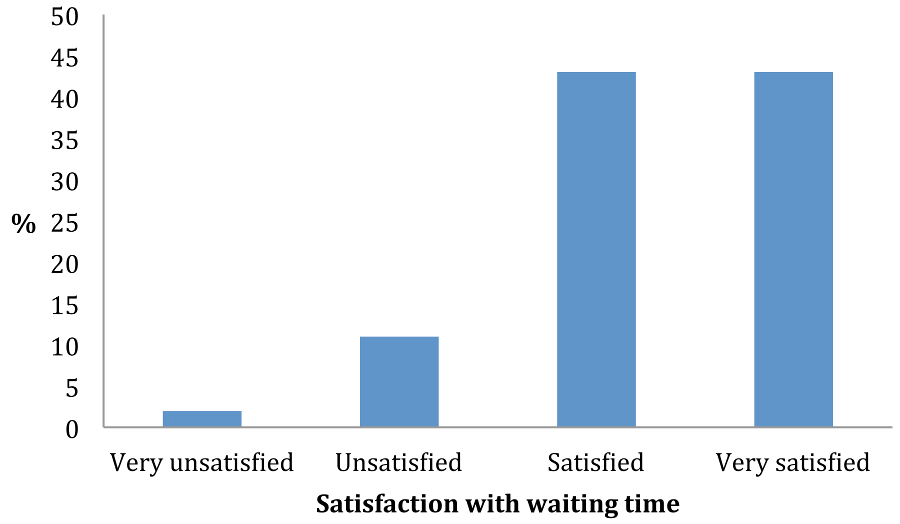

Figure 3: Satisfaction with the waiting time to be seen at the Joint Clinic

3. Knowledge and Expertise of the Joint Clinic Staff

*How satisfied were you with the **knowledge and expertise** of the Joint Clinic staff (physiotherapist and orthopaedic nurse)?*

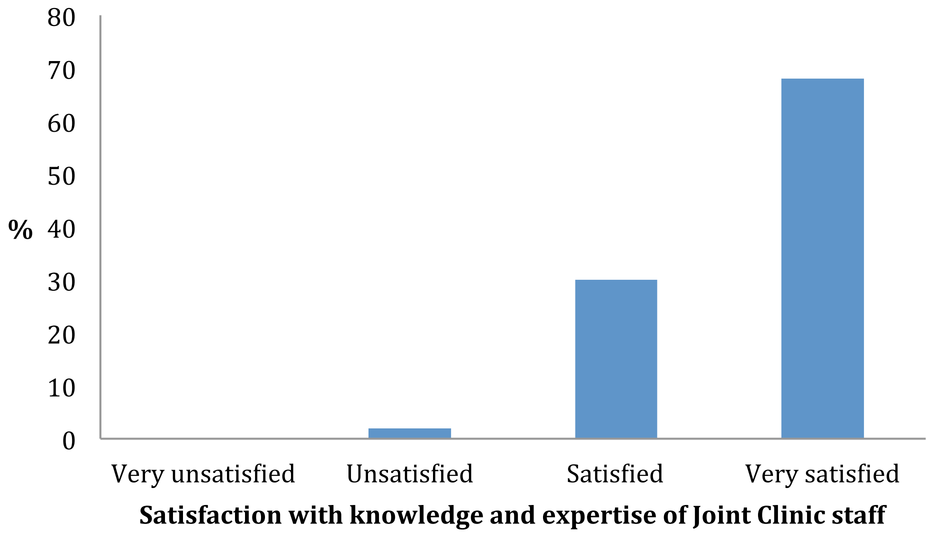

Figure 4: Satisfaction with the knowledge and expertise of Joint Clinic staff

4. Treatment by Joint Clinic staff

How satisfied were you with the ***advice, treatment, and plan of care*** *provided by the Joint Clinic staff (physiotherapist and orthopaedic nurse)?*

Missing data=2% (n=1)

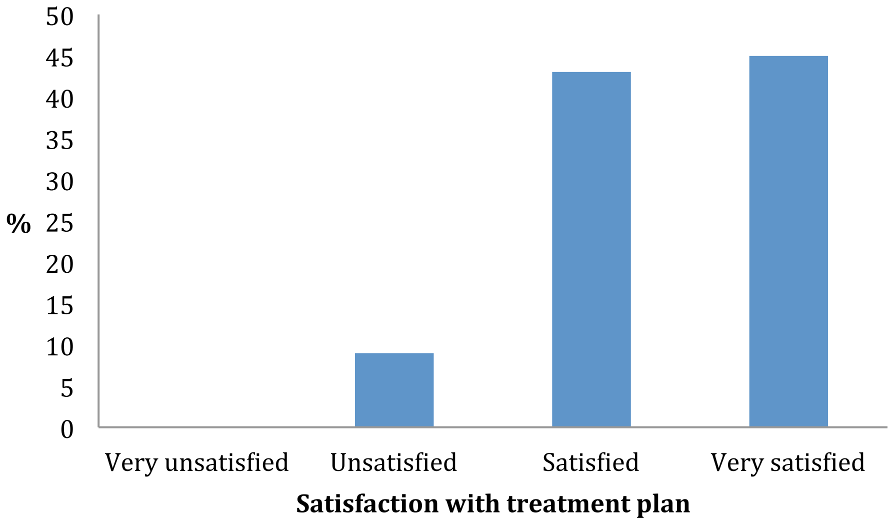

Figure 5: Satisfaction with the advice and treatment plan given by the Joint Clinic staff

5. Being seen by Joint Clinic staff rather than an Orthopaedic Surgeon

How satisfied were you to be seen by the Joint Clinic staff (physiotherapist and orthopaedic nurse) rather than an Orthopaedic Surgeon?

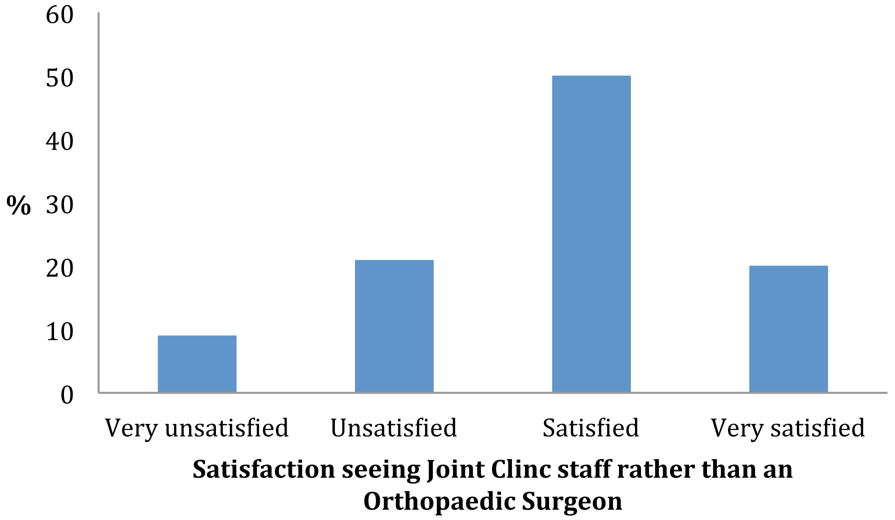

Figure 6: Satisfaction with being seen by Joint Clinic staff rather than an Orthopaedic Surgeon

6. Being seen by Joint Clinic staff if not offered an appointment with an Orthopaedic Surgeon at all

*If you knew that you would **not** be offered an appointment to see an Orthopaedic Surgeon **at all**, how satisfied would you be to have an appointment with the Joint Clinic staff (physiotherapist and orthopaedic nurse)?*

Missing data 2% (n=1)

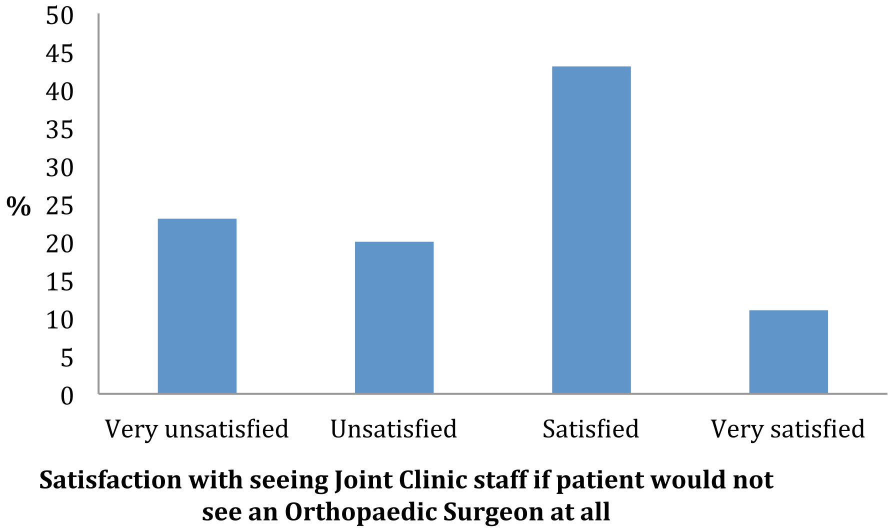

Figure 7: Satisfaction with seeing Joint Clinic staff if the patient would not be offered an appointment with an Orthopaedic Surgeon at all

7. Treatment at Physiotherapy Outpatients

*How satisfied were you with the **treatment and advice** provided by the <u>Outpatient Physiotherapy Department</u>, on the ground floor?*

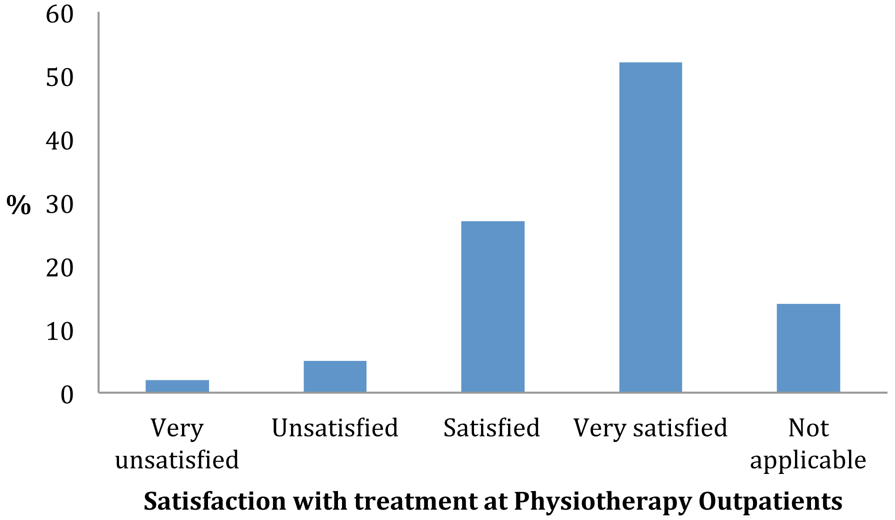

Figure 8: Satisfaction with treatment at Physiotherapy Outpatients

7a. Comments about treatment at Physiotherapy Outpatients (n=13)

Positive comments were made about the physiotherapy staff and the advice and treatment given. Physiotherapy staff were described with words such as “friendly,” “competent,” “excellent” and “wonderful.” Patients reported functional benefits such as improved mobility and decreased pain from the treatment provided and self-management strategies were appreciated. Several comments were negative.

Positive comments:

- “Very friendly and competent staff. Answered my questions adequately as they arose over the weeks.”
- “Both the individual sessions and the follow up group weekly course freed up the movement and flexibility of my knee and decreased the pain. Also gave me skills to continue on my own to maintain the improvement.”
- “Very satisfied, both with the 'one-on-one' sessions and the one hour group sessions.”
- “Very satisfied. Able to give lots of good advice. Excellent exercises. Gave good encouragement to attend the physio pool.”
- “My male physio was excellent. He was easy to talk with and always listened to any concerns and questions I had. He was sympathetic to the difficulties initially experienced with the exercises, offering solutions to make things easier until the muscles strengthened.”
- “Brilliant! 3 simple exercises which I still do daily and the hip pain is 95% better.”
- “Exercises and advice were a great help. Wished it went longer than eight weeks as some machines were really beneficial.”
- “[The physiotherapist] was excellent. She did everything she could to improve my situation.”
- “Being referred to an excellent physio who gave me exercises to help and treatment that gave me immediate relief of pain so that I can continue a reasonable standard of healthy activities.”
- “Have attended physio group classes, going ok. Staff wonderful.”

Negative comments:

- “No relief from knee pain but slight temporary ease in joint.”
- “One appointment [in] six month[s is] too long, how many six month[s] in my life[?]”
- “This was a waste of time…”

8. Other treatment

How satisfied were you with ***any other treatment and advice*** *arranged for you by the Joint Clinic staff? (E.g. brace, orthotics, dietician, social worker, and other referrals)*

Missing data 5% (n=2)

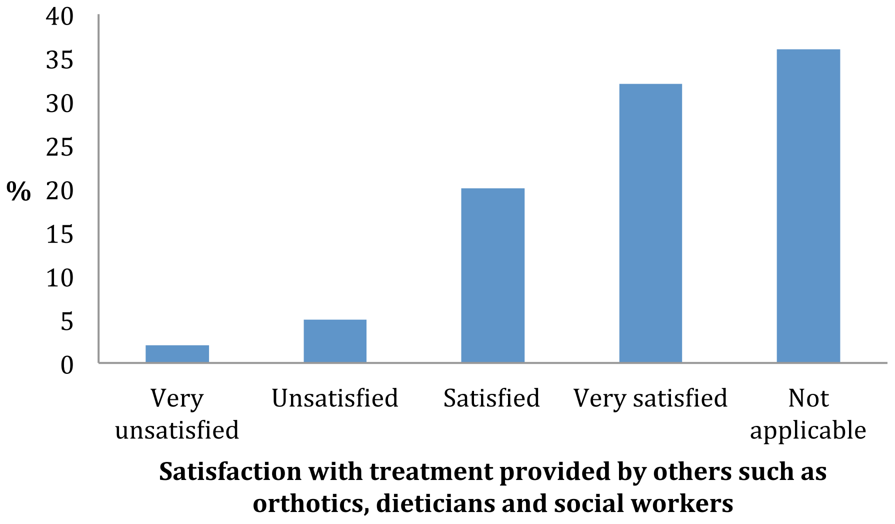

Figure 9: Satisfaction with treatment provided by others such as orthotics, dieticians and social workers

8a. Comments about treatment provided by others such as orthotics, dieticians and social workers (n=6)

- "[Very satisfied] if it was the right advice!"
- "This improved my comfort when walking markedly and overall improved my total mobility."
- "…very good small splint received which eases pain."
- "…an X-ray of the hip was requested by the clinician…"
- "Helped the pain when standing, getting into the car, household jobs etc."

One comment in this section was actually about physiotherapy but was external to that provided through the Joint Clinic:

- "I feel that the advice I was given by the physiotherapist to whom I was [referred] by my GP was every bit as good as that from the Joint Clinic Staff."

9. Main effect of the Joint Clinic

What has been the ***main effect*** *of attending the Joint Clinic for you?*

(n=30)

Patients reported that attending the Joint Clinic had led to functional improvements such as improved mobility, strength and general fitness. They felt they had received good advice and explanations and had an increased understanding of their condition having attended the Joint Clinic. They reported that attending the Clinic meant they were monitored and were part of the 'system.' Being able to see an Orthopaedic

Surgeon sooner than they would otherwise have been able to be was also noted as an effect of being seen in the Joint Clinic. Patients had increased coping mechanisms and self-management strategies. A reduction in pain and pain management was commented on. However, an increase in pain was also noted. Some patients felt there had been no effect. Psychological effects were commented on such as “hope” but also “depression.” The cost of physiotherapy appointments versus surgery and a return to work was questioned. Examples of quotes that illustrate these ideas are provided below:

Positive comments:

- “Getting more movement in knee.”
- “To have better mobility of joints with less pain.”
- “Understanding more about the condition.”
- “The feeling of not being completely ignored by the Health System.”
- “Coping strategies to manage pain, increase mobility and enable me to maintain fitness levels as far as possible.”
- “Far less pain in hip so now walking further and for longer. Feel 10 years younger and fitter. Not taking pain medication nearly as often.”
- “Hope and help to keep me mobile and independent.”

Negative comments:

- “Very painful right knee.”
- “None.”
- “…Our big question, was the cost of all the appointments, weeks of physio, pills etc, any cheaper than having surgery and GETTING BACK TO WORK!!!”

10. Overall outcome

How satisfied are you with the ***overall outcome*** *resulting from your visit to the Joint Clinic staff?*

Missing data 9% (n=4)

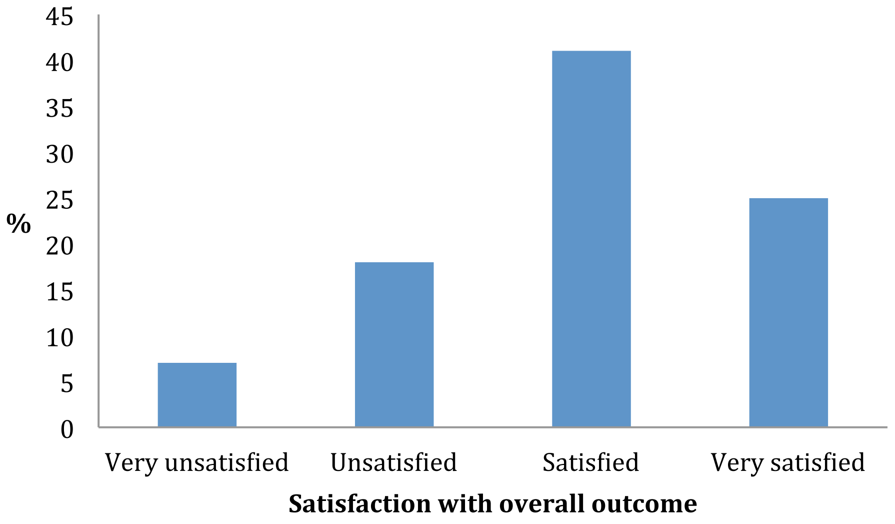

Figure 10: Satisfaction with the overall outcome of attending the Joint Clinic

10a. Comments regarding the overall outcome from attending the Joint Clinic (n=15)

Positive comments were that attending the Joint Clinic had given relief from pain and had kept people in 'the system.' The staff were also viewed positively. Some comments were neutral, or potentially positive but not definitively so, for example; " Regularly walking 3-5km without pain." Negative comments involved patients feeling little or no benefit from conservative treatment and that they required surgery and/or an appointment with an Orthopaedic Surgeon.

Positive or neutral comments:

- "Couldn't have asked for better help and by keeping up the exercises given I have gained some relief from the knee pain (which I am told is referred pain)."
- "Hopefully keeps me in the system as my work involves a lot of walking, lifting etc, of which I am finding at times extremely difficult and I do not want to retire yet!"
- "Helpful gap until seeing someone who can remedy my problem (before age catches up with me)."
- "I understand it is not badly worn enough yet to replace the joint."
- "I learnt that my R leg needs a further physio regimen."
- "I can seek to see an orthopaedic surgeon by asking my GP for a referral."

Negative comments:

- "The staff are very nice but it doesn't change my situation."
- "Although pain has continued and where it started in [the joint] it now also is [in another joint]."

- “I went into clinic with [a] very very sore aching knee joint and received no forcible relief.”
- “The pain from my hip still greatly compromises my everyday activities even though I take the maximum amount of paracetamol and diclofenac.”
- “I want a knee replacement.”
- “NOT because of staff. But what did we gain??”
- “I feel a person should be seen by an Orthopaedic Specialist for an appraisal. Obviously I am not ready to be operated on, but a professional diagnosis would be helpful.”
- “[One joint] already replaced. Would like [the other joint] replacement some time.”

11. Recommending Joint Clinic to others

Would you recommend the Joint Clinic to someone else in your situation?

Missing data 7% (n=3), ticked in between ‘no’ and ‘yes’ 2% (n=1)

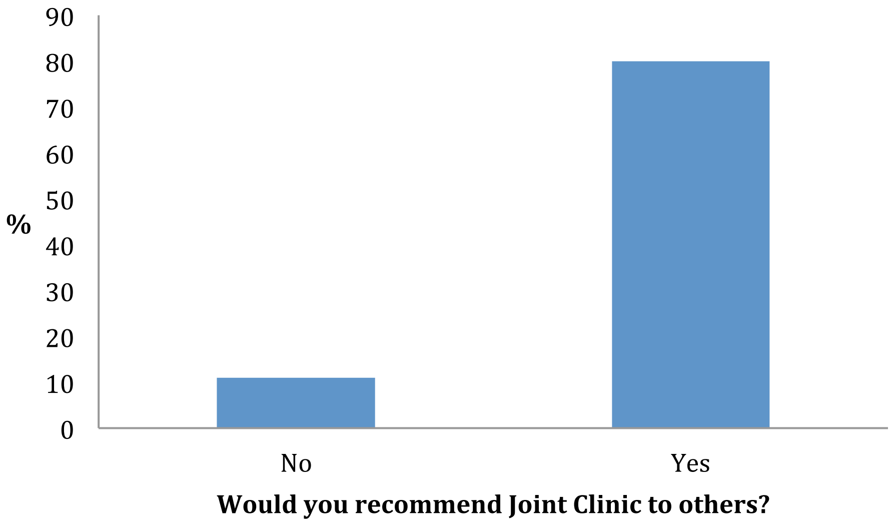

Figure 11: Recommending Joint Clinic to others

11a. Other comments about the Joint Clinic (n=20)

Some of these comments were not directly about the Joint Clinic (for example thanking the researchers for providing a pen with the survey). Others were just noting aspects such as the particular place they had attended physiotherapy, for example Mosgiel. More substantial comments involved positive aspects such as the staff listening and allowing the patient time to discuss aspects including wider health issues. The manner of the staff was complimented and staff were thanked by patients. It was raised that more frequent follow-up appointments (the patient suggested 3 monthly) may be beneficial. Negative aspects involved patients wanting to have surgery and an appointment with an Orthopaedic Surgeon. It was commented that there was no physiotherapist at the Physiotherapy pool and that there was a lack of clarity about who they would be seeing at the Joint Clinic.

Positive comments:

- "Nothing to say except keep up the good work and thank you for your time and patience."
- "I appreciated the way staff really listened and understood what I was saying in my 'layman's terms.' Also attended to my 'whole' well-being e.g. mood etc, general health."
- "Very polite and courteous. Arranged X-rays and following appointments."
- "I think the Joint Clinic is a very valuable service, gives you time to discuss matters re your situation more thoroughly: Helps you to maintain your mobility as best you can."
- "With the follow-up appointment in 6 months, I have the opportunity to reassess the [joint] and have professional service with physio etc ongoing. This will also give me access to a surgical opinion if needed"
- "Could be helpful to some people"
- "Probably not possible but 3 monthly visits to the Clinic would be better than 5 monthly…Personally I feel that the clinic, its clinicians and nurses have helped make a big difference with their diagnosis and referrals and their place in the orthopaedic process is very important."
- "Many thanks to the staff and … the physio."

Negative comments (some are mixed positive and negative)

- "Look at the age of patients before taking up physiotherapist's time."
- "Other than referral to the physiotherapist then to the pool which I found very good but I feel that I would like to have seen the orthopaedic surgeon."
- "I was advised that the physio pool may help me and it does, but I feel that it would be better if someone there could tell me what to do. There are no physiotherapists there."
- "When I went for my first appointment I thought I was being seen [by an] Orthopaedic Surgeon and did not realise it was [a] Physiotherapist or Orthopaedic Nurse..."
- "I refer to letter … recommending surgery."
- "I do hope that I can get the pain relieved/removed in the near future."
- "[Given conflicting opinions] I feel therefore that I need to get an opinion from an orthopaedic surgeon… I would like an assessment from an orthopaedic surgeon to … [discuss] how far away an operation is likely to be … I feel GPs could be encouraged more to send patients to the joint clinic…"

DISCUSSION

In this study, 82% of participants reported that their overall experience of attending the Joint Clinic was 'good' or 'excellent.' The majority of participants were 'satisfied' or 'very satisfied' with the time they waited to be seen at the Clinic (86%), the knowledge and expertise of Joint Clinic staff (98%), the treatment plan given by Joint Clinic staff (89%), their treatment at Physiotherapy Outpatients (92% of those this applied to) and other treatment provided such as through orthotics or dieticians (82% of those this applied to). Most participants were 'satisfied' or 'very satisfied' to be seen by Joint Clinic staff rather than an Orthopaedic Surgeon (70%). However, if patients knew that they would not be seen by an Orthopaedic Surgeon at all, fewer patients would be 'satisfied' or 'very satisfied' to be seen by Joint Clinic staff (55%). Only 68% of participants responded to the open ended question about the main effect of attending the Joint Clinic. Of those that responded, both positive and

negative effects were noted. Positive effects included improved functioning, strength and fitness, reduced pain and an increased understanding and ability to self-manage their condition. Negative effects included feeling that there had been no effect of attending the Joint Clinic. However, 66% of participants were ‘satisfied’ or ‘very satisfied’ with the overall outcome resulting from attending the Joint Clinic and 80% would recommend the Joint Clinic to others in their situation.

The free text ‘comments’ were only completed by a small percentage of participants. Comments were often negative, although positive comments were also provided. However, considering the information shown in the histograms, which relate to questions completed by most, if not all, participants, a markedly more favourable impression is given. This may indicate that those with strongly negative views may have been more motivated to provide free text comments. Negative comments included ideas that those patients wanted to have surgery, to be seen by an Orthopaedic Surgeon or that they had found no benefit from attending the Joint Clinic. It may be that there are particular patients for whom the Joint Clinic will not be effective and/or who would benefit from seeing an Orthopaedic Surgeon. It therefore seems important to ensure that appropriate patients are being seen at the Joint Clinic and also that those patients seen at the Joint Clinic who are not likely to benefit and should be seen by an Orthopaedic Surgeon, are referred on. It may be beneficial to ensure that patients coming to the Joint Clinic are aware of purpose of the Clinic but also that they are aware of the alternatives that could be provided to them under the current system if the Joint Clinic was not an option for them. For example, the alternatives may not be surgery or being seen at the Joint Clinic, they may be being referred back to their GP or being seen in the Joint Clinic.

A strength of this study was the high response rate (71%). However, despite this, there were limited numbers involved due to the relatively small number of patients that had had a follow-up appointment at the Joint Clinic at the time of the study. Satisfaction with a range of aspects of the Joint Clinic was investigated and participants had the opportunity to provide free text comments to explain their responses or to raise aspects not specifically asked in the survey.

CONCLUSION

Overall, the findings were very positive with the majority of participants ‘satisfied’ or ‘very satisfied’ with the aspects examined. Of note, was the relatively lower level of satisfaction that participants reported that they would have if they knew they would not be seen by an Orthopaedic Surgeon at all. Some people found no benefit from attending the Clinic and attempting to accurately assess those patients who may (or may not) benefit from the Joint Clinic both in the referral process and when seen in the Clinic may be important.

APPENDICES END
